# Effect of E-Beam and X-Ray Irradiation on Radiation–Chemical Yield and Reaction Rate of Volatile Organic Compound Transformations

**DOI:** 10.3390/molecules30214226

**Published:** 2025-10-29

**Authors:** Victoria Ipatova, Ulyana Bliznyuk, Polina Borshchegovskaya, Timofey Bolotnik, Alexander Chernyaev, Igor Gloriozov, Elena Kozlova, Alexander Nikitchenko, Anastasia Oprunenko, Mariya Toropygina, Irina Ananieva, Igor Rodin

**Affiliations:** 1 Skobeltsyn Institute of Nuclear Physics, Lomonosov Moscow State University, GSP-1, 1-2 Leninskiye Gory, 119991 Moscow, Russia; uabliznyuk@gmail.com (U.B.); alexeevapo@mail.ru (P.B.); a.p.chernyaev@yandex.ru (A.C.); 2 Department of Physics, Lomonosov Moscow State University, GSP-1, 1-2 Leninskiye Gory, 119991 Moscow, Russia; nikitchenko.ad15@physics.msu.ru; 3 Department of Chemistry, Lomonosov Moscow State University, GSP-1, 1-3 Leninskiye Gory, 119991 Moscow, Russia; timab@tut.by (T.B.); gloriozov@nmr.chem.msu.ru (I.G.); oprunenko_anastasiya@mail.ru (A.O.); irishan@mail.ru (I.A.); igorrodin@yandex.ru (I.R.); 4 Department of Medical and Biological Physics, Federal State Autonomous Educational Institution of Higher Education I.M. Sechenov First Moscow State Medical University of the Ministry of Health of the Russian Federation (Sechenovskiy University), 8-2 Trubetskaya str., 119991 Moscow, Russia; waterlake@mail.ru (E.K.); tim.mmit@yandex.ru (M.T.); 5 Lomonosov Institute of Fine Chemical Technologies, MIREA—Russian Technological University, 78 Vernadsky Ave., 119571 Moscow, Russia

**Keywords:** biological objects irradiation, electron beam, X-rays, volatile organic compound, 1-hexanol, gas chromatography-mass spectrometry, radiation–chemical yield, density functional theory (DFT)

## Abstract

This study investigates the impact of 1 MeV electron beam and 80 keV X-ray irradiation on the decomposition rate and radiation–chemical yield of 1-hexanol in aqueous saline solution to develop a comprehensive approach to determining reliable volatile organic compound markers for food irradiation. A 50 mg/L 1-hexanol solution was irradiated with the doses ranging from 100 to 8000 Gy at various dose rates ranging from 0.2 to 10 Gy/s to assess the impact of irradiation parameters on the decomposition rate and radiation–chemical yield of volatile compounds typically found in food. GC–MS analysis revealed a non-linear decrease in 1-hexanol concentration with increasing dose, accompanied by the formation of aldehydes, ketones, and secondary alcohols. Among these products, hexanal was detected at the lowest applied dose and exhibited dose-dependent behavior that correlated strongly with 1-hexanol degradation. Density functional theory calculations identified the most probable pathways for the formation of hexanol decomposition products, involving direct ionization, radical reactions, and oxidation. A mathematical model proposed in the study describes dose-dependent transformations of 1-hexanol into hexanal, enabling quantitative estimation of the degradation extent of hexanol. The findings suggest that hexanal can serve as a quantitative marker for hexanol degradation, supporting the development of rapid “dose range” determination methods for food irradiation that ensure microbial safety while minimizing undesirable oxidation of proteins, fats, and carbohydrates.

## 1. Introduction

Over the recent years radiation technologies have been successfully applied in a wider range of areas encompassing material modification, medicine, environmental safety, goods handling and the food industry [1,2,3,4]. A great variety of applications and complexity of the novel irradiation objects call for optimization of irradiation parameters to maximize the desired effect without causing a negative impact on the irradiated object [5,6]. Compared to thermal processing, irradiation increases the temperature of food products only marginally, which makes it applicable to a wider range of food products [7]. Currently, electron accelerators are considered to be most effective for industrial irradiation since this high-performance technology allows to reduce processing time, which is essential for large-scale production, and vary the penetration depth of electrons depending on the irradiation goal. To enable a smooth integration of irradiation technology in food manufacturing industries, low-energy electrons and bremsstrahlung irradiation have been extensively used for surface treatment of foods that are particularly sensitive to irradiation [8].

When foods and other biological objects are exposed to ionizing radiation, different physical and chemical processes are initiated by direct ionization of biomolecules and atoms of irradiated bio-objects and indirect action through reactive oxygen species that trigger radiation-induced chemical reactions involving biomolecules [9,10]. Reactive products occurring as a result of water radiolysis, such as hydroxyl radicals (•OH), hydrated electrons (e^−^_aq_), and hydrogen atoms (H•), interact with biomolecules, initiating a cascade of radiation-induced chemical transformations, including oxidation of molecules, bond breaking, and structural rearrangements.

Being highly sensitive to any physical and chemical factors affecting bio-objects, low-molecular weight volatile organic compounds (VOCs) can serve as indicators of the degree of the impact of irradiation on biological objects. In the food industry, 2-alkylcyclobutanones (2-ACBs), such as 2-dodecylcyclobutanone and 2-tetradecylcyclobutanone can be regarded as specific irradiation markers as they occur as a result of triglyceride radiolysis in fat-containing food products and are not produced by thermal or oxidative degradation [11]. In medicine, VOCs can be used as potential biomarkers to diagnose the health status of a patient and detect various illnesses, including cancer and infectious diseases [12,13,14]. In ecology, VOCs can provide information on environmental conditions, pollution levels, and biological responses to stressors caused by industrial activities or decomposition of organic matter [15,16].

The largest area of application of volatile organic compounds, however, is the food industry, where VOCs are essential markers of a product’s quality and safety. They reveal enzymatic activity of a large number of pathogenic bacteria and fungi [17,18,19,20] and are also used for detecting product oxidation and spoilage during long-term storage [21,22,23,24]. In agriculture, VOCs can serve as indicators of residual pesticides or other chemicals used to treat agricultural products and plants when they are planted and grown [25]. Gas chromatography coupled with mass spectrometry (GC-MS) is currently the gold standard method for VOC analysis, as it enables the detection and quantification of hundreds of different compounds. Although GC-MS provides high sensitivity and selectivity [26,27], its effectiveness can be influenced by the complexity of biological matrices, including factors such as matrix effects, co-elution of compounds, and thermal degradation of thermolabile analytes [28,29,30].

Some volatile organic compounds typically found in foods are particularly sensitive to any physical and chemical changes that may occur in the product during long-term storage after it has been subjected to thermal or non-thermal processing [23,31]. Aldehydes, for instance, are sensitive to the dose absorbed by the food product during irradiation as they are oxidation products of fats, proteins, and carbohydrates [32,33,34]. The presence of alcohols in food products detected during storage, on the other hand, is a clear indicator of bacterial activity in the irradiated foods [35,36,37]. The presence of oxygen during irradiation enhances radical chain reactions and contributes to more extensive radiation-induced molecular damage in foods, primarily due to the formation of peroxyl radicals and oxidative degradation products [38,39]. It should be noted that antioxidants, such as ascorbate or tocopherols, can bind reactive radicals and inhibit the formation of VOCs [40]. However, irradiation can also affect the antioxidants, which reduces the antioxidant effect on organic molecules [41]. Another factor influencing reaction kinetics of the VOCs formation and degradation is temperature. An increase in temperature causes the mobility of radicals to increase which leads to a higher chemical reaction rate, affecting both the yield and the spectrum of VOCs formed after irradiation [42,43].

Food irradiation planning can benefit from the use of VOCs for determining optimal dose ranges and physical parameters which are able to enhance food irradiation efficiency. The optimal dose range which varies depending on the composition of the food item and initial bacteria contamination, lies between the dose that cannot guarantee the suppression of bacteria throughout the product and the dose that triggers intensive lipid and protein oxidation causing an undesired change in the taste and smell of the irradiated product [5].

It should be noted that radiosensitivity of different bacteria and biomacromolecules depends not only on the type of microorganisms and molecular structure but also on physical irradiation parameters, such as the type of irradiation, its energy spectrum and the irradiation dose rate [44,45,46]. Since VOCs can serve as criteria for determining optimal dose ranges for different categories of food products, it is important to understand how the dose limits would change depending on physical irradiation parameters.

The objective of the study is to develop a novel approach to determining food irradiation markers among volatile organic compounds. In the absence of the criteria for establishing optimal irradiation dose ranges to meet different irradiation goals while minimizing a negative impact on food quality, we have researched the radiation–chemical yield of VOCs to find the potential markers whose concentration predictably changes after irradiation and can, therefore, serve as quantitative markers for food irradiation. For this purpose, we have performed a series of model experiments involving irradiation of hexanol solutions with low-energy electron beam (e-beam) irradiation and bremsstrahlung irradiation within the dose range from 100 Gy to 8000 Gy spanning different irradiation goals, such as sprouting control (up to 1 kGy) and expanding the shelf life of food products by inhibiting pathogenic bacteria (3–7 kGy) [5,47]. The study presents our findings on chemical transformations and the reaction rate of hexanol, which is one of the most frequently identified VOCs typically occurring in food products [23,48,49,50,51,52,53,54,55]. 1-Hexanol can be found in various food matrices, including meat [48,53], fish [55], dairy products [51], and plant-derived materials [50,52]. It can be formed both as a result of enzymatic activity of microorganisms [52] and as a primary product of lipid degradation during storage [56] or under the influence of irradiation, particularly in products containing unsaturated fatty acids [23,31,57]. We selected 1-hexanol among other VOCs typically found in food products as a model molecule for studying the radiation-induced transformations of low-molecular-weight compounds. Since 1-hexanol is a primary degradation product of unsaturated fatty acids, studying transformations of hexanol alcohol in model solutions allows to extend possible degradation mechanisms of nutrients and VOCs to real food matrices.

The specified dynamics of the decomposition of hexanol and its decomposition products allowed us to find the high-probability marker which is typical for hexanol decomposition. A similar approach can be taken to determine the irradiation markers among volatile compounds which are decomposition products of proteins, fats, and carbohydrates. Knowing the dose dependencies of VOCs irradiation markers can help to establish specific doses which prevent any undesirable physical and chemical modifications to the food product. Since industrial irradiation facilities use e-beam irradiation and X-ray irradiation at different dose rates, the study compares the VOCs yield and hexanol decomposition rate in hexanol solution irradiated with 1 MeV accelerated electrons and 80 keV X-rays which can be used for surface irradiation of foods.

## 2. Results and Discussion

### 2.1. Research Sequence

**Figure 1:**
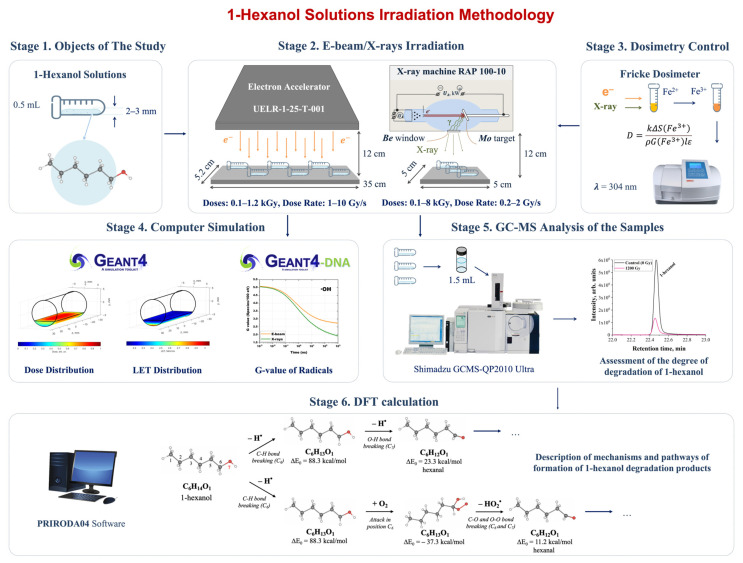
Research stages: (**1**) preparation of 1-hexanol solution; (**2**) irradiation of the samples with accelerated electrons and X-rays; (**3**) Fricke dosimetry control; (**4**) Geant4 and Geant4-DNA computer simulation; (**5**) GC-MS analysis; (**6**) DFT calculations.

The study of the impact of irradiation on the chemical yield and reaction rate of volatile organic compound transformations involved six stages (Figure 1) which are described in the materials and methods section.

At the first stage, 1-hexanol alcohol was diluted in a 0.9% saline solution to the initial concentrations of 50 mg/L and placed in polypropylene 2 mL Eppendorf tubes. At the second stage, a part of the 1-hexanol solution was irradiated with 1 MeV electrons and the other part was exposed to 80 keV bremsstrahlung irradiation. The irradiation dose and the dose rate applied to the hexanol solution varied to observe the dynamics of 1-hexanol decomposition and accumulation of VOCs resulting from 1-hexanol decomposition. The 50 mg/L 1-hexanol solutions were irradiated with accelerated electrons with the doses ranging from 100 Gy to 1200 Gy at the dose rates of 1 Gy/s and 10 Gy/s, while X-ray irradiation was performed with the doses ranging from 100 Gy to 2000 Gy at the dose rate of 0.2 Gy/s and with the doses ranging from 100 Gy to 8000 Gy at the dose rate of 2 Gy/s.

The third stage involved measuring the doses absorbed by the 1-hexanol solution during electron beam irradiation and X-ray irradiation using Fricke dosimetry solution. At the fourth stage, we performed Geant4 computer simulation to control the dose uniformity in the irradiated solutions. Geant4-DNA computer simulation was carried out to compare the contribution of direct ionization and indirect action through free radicals during e-beam irradiation and X-ray irradiation.

Stage five, which followed immediately after irradiation with e-beam or X-rays, involved GC-MS analysis to measure the concentration of 1-hexanol and its decomposition products in irradiated and non-irradiated 1-hexanol solutions. The impact of the irradiation dose and the dose rate on the hexanol decomposition rate and accumulation rate of 1-hexanol decomposition products was estimated separately for e-beam and X-ray irradiated solutions. We also compared the 1-hexanol decomposition rate after the 1-hexanol solution was irradiated with e-beam and X-rays at similar dose rate values. At the sixth stage, we found the key quantitative indicator of the extent of 1-hexanol decomposition using a mathematical model of the dynamics of VOCs and mechanisms behind radiation-induced VOCs transformations in the irradiated solutions calculated based on density functional theory (DFT) using Priroda04 software.

### 2.2. The Impact of Irradiation Dose and the Dose Rate on the Transformation of 1-Hexanol into Its Degradation Products

**Figure 2:**
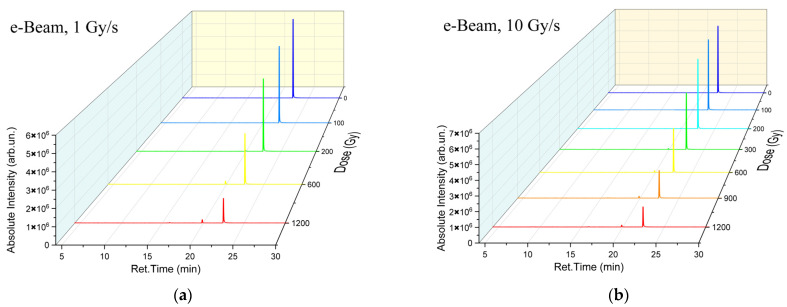

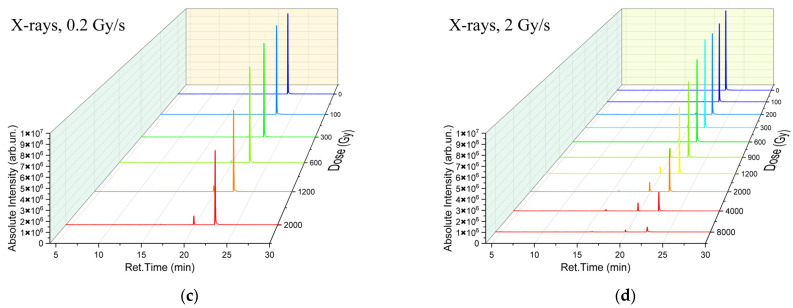
Chromatograms of non-irradiated 1-hexanol suspension samples and the samples irradiated with e-beam at the dose rates 1 Gy/s (**a**) and 10 Gy/s (**b**) as well as the samples irradiated with X-rays at the dose rates 0.2 Gy/s (**c**) and 2 Gy/s (**d**).

To illustrate the impact of accelerated electrons and X-rays on the concentration of 1-hexanol and VOCs which appeared as a result of 1-hexanol degradation in the irradiated suspensions we have provided the chromatograms of the non-irradiated 1-hexanol suspension as well as the suspensions irradiated with accelerated electrons (Figure 2a,b) and X-rays (Figure 2c,d) at different doses and dose rates. As it can be seen from Figure 2a–d, both irradiation types decreased the concentration of 1-hexanol and led to the formation of 1-hexanol degradation products.

**Figure 3:**
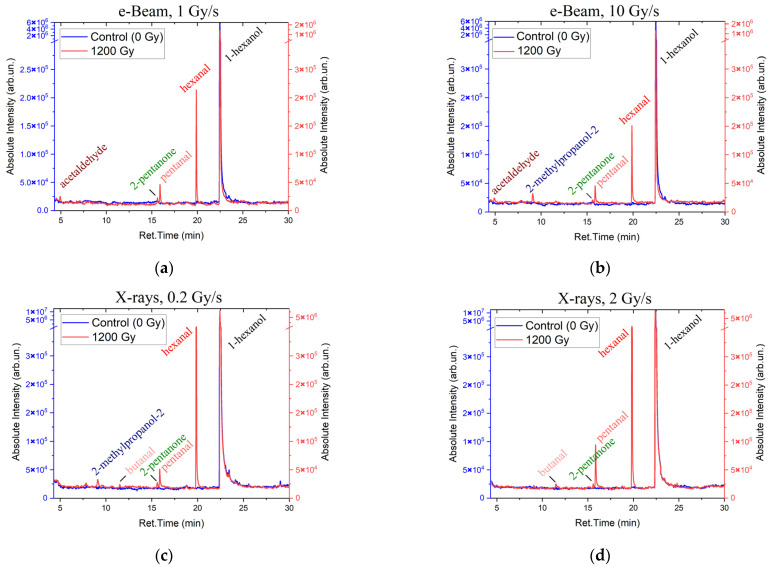
Chromatograms of non-irradiated 1-hexanol suspension samples and the samples irradiated with 1200 Gy. The blue lines show the non-irradiated 1-hexanol suspension sample. The red lines show the samples irradiated with accelerated electrons at the dose rates 1 Gy/s (**a**) and 10 Gy/s (**b**) as well as the samples irradiated with X-rays at the dose rates 0.2 Gy/s (**c**) and 2 Gy/s (**d**).

To compare the concentrations of 1-hexanol and the VOCs which appeared as a result of 1-hexanol degradation in the suspensions irradiated at different dose rates, Figure 3 shows the 1-hexanol suspension irradiated with the dose of 1200 Gy at the dose rates 1 Gy/s (Figure 3a) and 10 Gy/s (Figure 3b) for electrons and 0.2 Gy/s (Figure 3c) and 2 Gy/s (Figure 3d) for X-ray irradiation as well as the non-irradiated 1-hexanol suspensions.

A total of six compounds were identified in the 1-hexanol suspension irradiated with accelerated electrons; alcohols: 1-hexanol and 2-methylpropanol-2; a ketone: 2-pentanone; and aldehydes: acetaldehyde, pentanal, and hexanal. In the samples irradiated with X-rays, six compounds were also identified in the 1200 Gy suspensions, and ten compounds were detected at the dose of 8000 Gy; alcohols: 1-hexanol and 2-methylpropanol-2; ketones: acetone, 2-butanone, and 2-pentanone; and aldehydes: acetaldehyde, propanal, butanal, pentanal, and hexanal. All identified compounds, their molecular weights, retention times, and concentrations are given in Table A1, Table A2, Table A3 and Table A4 in Appendix A. It can be assumed that the higher the dose the more VOCs appearing as a result of 1-hexanol degradation were identified in the 1-hexanol solution.

**Figure 4:**
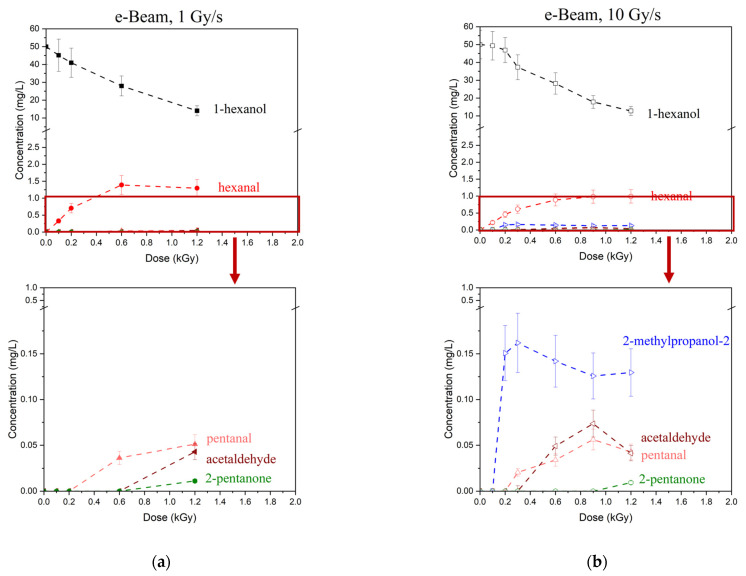

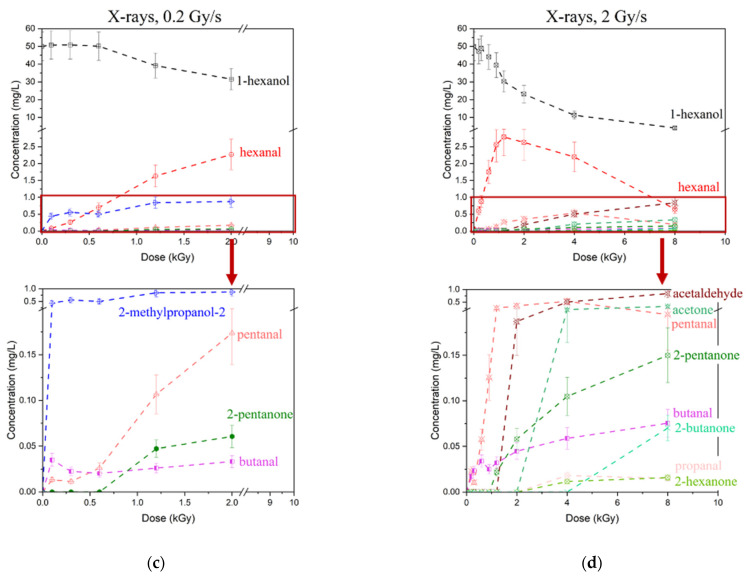
Dependencies of 1-hexanol concentration and its decomposition products on the irradiation dose when irradiated with accelerated electrons at the dose rate 1 Gy/s (**a**) and 10 Gy/s (**b**) and with X-ray irradiation at 0.2 Gy/s (**c**) and 2 Gy/s (**d**).

Figure 4a–d shows the dependencies of 1-hexanol concentration on the irradiation dose and the dependencies of the concentrations of VOCs formed due to the decomposition of 1-hexanol. While most VOCs were present in irradiated 1-hexanol solutions in the marginal concentration of less than 1 mg/L, hexanal was the main contributor to the radiation–chemical yield of 1-hexanol molecules. It should be noted that the irradiation with doses up to 2000 Gy increased the hexanal concentration; a further increase in the dose, however, decreased the hexanal concentration. An initial increase in the concentration of 1-hexanol degradation products with a higher dose range followed by the decrease in their concentration is an indication of two competing processes: the accumulation of degradation products due to 1-hexanol decomposition as well as the decomposition of degradation products as a result of irradiation.

After irradiation most VOCs were detected with a certain threshold dose D_T_ exceeding 100 Gy, while hexanal was identified at 100 Gy and higher. An increase in the dose rate decreased the threshold dose D_T_ for most VOCs identified in 1-hexanol after irradiation with both accelerated electrons and X-rays. These results align with the study showing that the degradation pathways of benzene and dienes under the action of irradiation strongly depend on the dose rate [54].

To compare the impact of accelerated electrons and X-rays on the decomposition rate of hexanol and hexanal accumulation rate in hexanol solutions irradiated with the doses ranging from 100 Gy to 2000 Gy at dose rates ranging from 0.2 Gy/s to 10 Gy/s we performed a two-way and a three-way ANOVA test. The test showed a significant difference between the 1-hexanol decomposition rates in the solutions irradiated with X-ray irradiation and electron irradiation (*p*-value = 0.00027). The comparison of the 1-hexanol decomposition rate under the action of e-beam and X-ray irradiation at close dose rates, equal to 1 Gy/s for electrons and 2 Gy/s for X-rays, showed that the 1-hexanol decomposition rate was significantly higher (*p*-value = 0.011) for e-beam irradiation compared to X-rays at the doses ranging from 300 Gy to 2000 Gy.

The accumulation rate of hexanal was significantly higher (*p*-value = 0.006) when the 1-hexanol solution was irradiated with X-rays compared to e-beam and depended on the dose rate for doses ranging from 300 Gy to 2000 Gy. Interestingly, while a ten-fold increase in the X-ray dose rate led to a marginal increase in the hexanal accumulation rate (*p*-value = 0.10), no significant difference was observed for electron beam irradiation between the dose rates of 1 and 10 Gy/s (*p*-value = 0.49) in the dose range of 300 Gy to 1000 Gy.

### 2.3. Absorbed Dose Distribution, Linear Energy Transfer (LET) Distribution and Radiation–Chemical Yield of Free Radicals in Water Irradiated with Electron Beam and X-Rays

**Figure 5:**
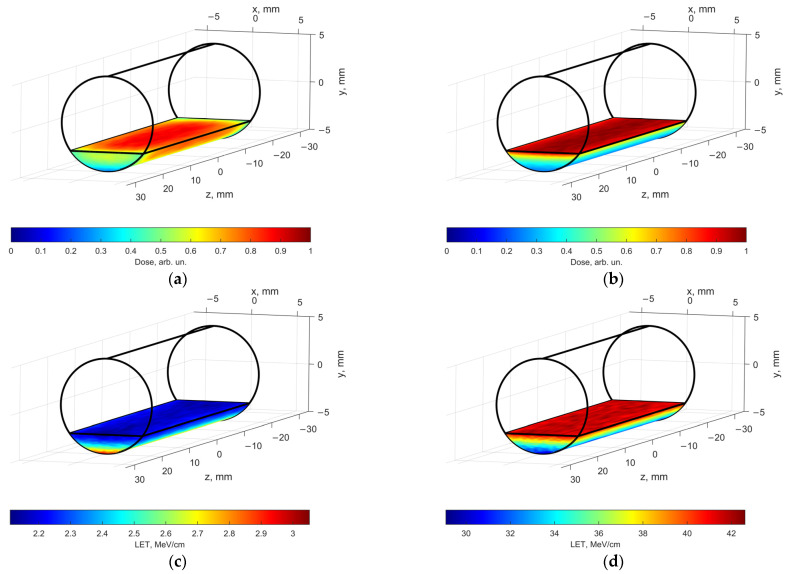
Dose distribution (**a**) and LET distribution (**c**) in the water phantom when it is exposed to 1 MeV accelerated electrons; dose distribution (**b**) and LET distribution (**d**) in water phantom irradiated with 80 keV X-rays.

To assess the contribution of direct ionization and indirect action of irradiation to radiation-induced transformations of hexanol, we performed Geant4 and Geant4-DNA computer simulation which is used to calculate distributions of dosimetry values and radiation–chemical yield of free radicals for e-beam irradiation and X-ray irradiation. The simulation uses energy spectra of accelerated electrons (Section 3.2) and X-rays (Section 3.2) as well as the linear dimensions of polypropylene tubes filled with hexanol solutions, specifically for each irradiation method. Figure 5 shows the calculated spatial absorbed dose distributions generated by a 1 MeV electron beam (Figure 5a) and by 80 keV X-rays (Figure 5b) in a 2 mm thick water phantom simulating a solution placed in a polypropylene cylindrical Eppendorf tube. The geometry of the phantom corresponds to the geometry of the solution in a 2 mL Eppendorf tube.

When the water phantom is irradiated with 1 MeV accelerated electrons (Figure 5a), the dose uniformity, which is the ratio of the minimum to the maximum dose, is 0.3 in the entire volume of the phantom with most of the volume having the dose uniformity of 0.6. Similarly, when the water phantom is irradiated with 80 keV X-ray photons (Figure 5b), the dose uniformity is slightly under 0.3. Compared with accelerated electrons, the absorbed dose generated by X-rays decreases more sharply with depth, which is associated with an exponential weakening of the radiation fluence when passing through the water phantom.

Linear energy transfer (LET) values averaged over the absorbed energy in the water phantom irradiated with accelerated electrons (Figure 5c) are much lower (~2.2–3.0 MeV/cm) compared with LET of secondary electrons formed as a result of the interaction of X-rays (Figure 5d) with the water phantom (up to 42 MeV/cm). In the case of X-ray irradiation, more ionization events are formed per unit path of secondary electrons passing in the water than per unit path of 1 MeV primary electrons.

Despite the dose non-uniformity and the different nature of the interaction of electrons and X-rays with water, the use of water solutions provides partial equalization of concentrations of free radicals occurring as a result of water radiolysis due to diffusion and convection, especially when linear dimensions of the volume are only several milliliters.

**Figure 6:**
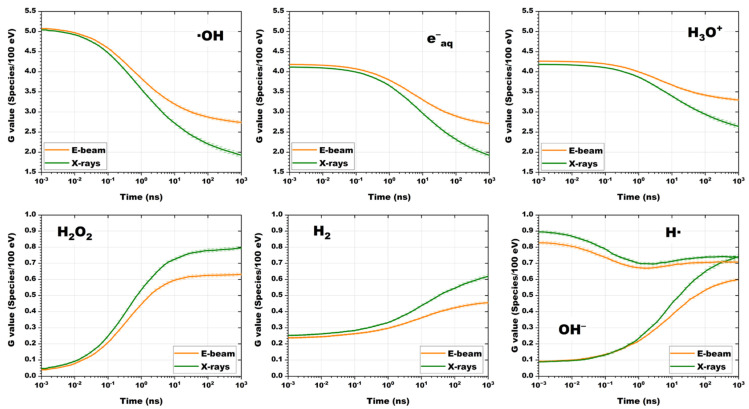
The dependencies of G-values for radicals formed as a result of e-beam irradiation (orange curve) and X-ray photons (green curve) on time.

The calculated time dependencies of the radiation–chemical yield of free radicals, G (species/100 eV), caused by the exposure to 1 MeV accelerated electrons and 80 keV X-ray photons are shown in Figure 6.

It can be seen from Figure 6 that when the water is exposed to 1 MeV accelerated electrons and 80 keV bremsstrahlung photons, the changes in the radiation–chemical yield of radicals over time have similar dynamics. The radiation–chemical yield of •OH, e^−^_aq_, H_3_O^+^ and H• radicals for both types of irradiations have close values after exposure during 10^−3^–1 ns. As the time passes, from 1 ns to 10^3^ ns, the G-values in X-ray irradiated water decrease faster than in the water irradiated with accelerated electrons, which is probably due to the rapid recombination of radicals in areas of high ionization density. The rate of accumulation of H_2_O_2_, H_2_ and OH^−^ during X-ray irradiation is higher, especially during the time exceeding 10 ns, which can be due to high LET values which can lead to frequent “radical-radical reactions”, such as •OH + •OH → H_2_O_2_ and H• + H• → H_2_.

According to Figure 6, the calculated G-values (species/100 eV) for all radicals registered at 10^3^ ns are:

$$\left(1\;MeV\;e^{-}\right)⇝H_{2}O\to •OH\left(2.7\right)+e_{aq}^{-}\left(2.7\right)+H_{3}O^{+}\left(3.3\right)+•H\left(0.71\right)+H_{2}O_{2}\left(0.63\right)+H_{2}\left(0.46\right)+{OH}^{-}(0.60)$$

$$\left(80\;keV\;\gamma \right)⇝H_{2}O\to •OH\left(1.9\right)+e_{aq}^{-}\left(2.7\right)+H_{3}O^{+}\left(2.6\right)+•H\left(0.74\right)+H_{2}O_{2}\left(0.80\right)+H_{2}\left(0.62\right)+{OH}^{-}(0.74)$$

The calculated G-values of various radicals when the water is exposed to accelerated electrons fully correspond to similar experimental values, practically do not depend on the dose rate, and are within the measurement errors described in [58], which indicates the reliability of the simulation. It can be concluded that the type of irradiation and its energy spectrum determine not only the spatial dose distribution, but also the spatial distribution of water radiolysis products. These differences should be taken into account when interpreting the results of radiation–chemical transformations of 1-hexanol and its decomposition products presented in this work.

The formation of radicals is a very rapid process occurring during irradiation and ranging from a few nanoseconds to a few microseconds. Since degradation products of hexanol were detected in hexanol solution immediately after irradiation, it can be concluded that all transformations of hexanol and its degradation products occurred during irradiation.

### 2.4. Comparison of Radiation-Induced Degradation Efficiency of 1-Hexanol After Electron Beam Irradiation and X-Ray Irradiation

**Figure 7:**
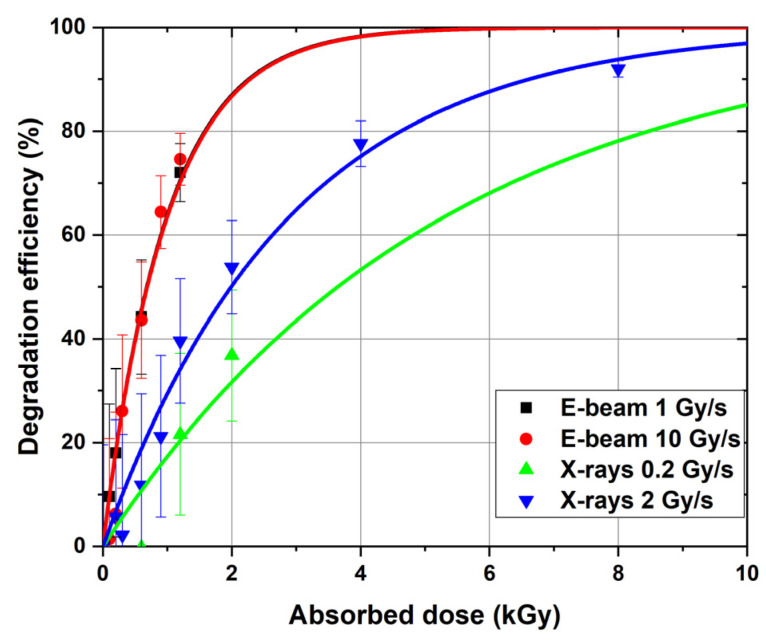
Dose dependencies of the degradation efficiency of 1-hexanol after exposure to accelerated electrons at the dose rate of 1 Gy/s (black dots) and 10 Gy/s (red dots), and X-rays at the dose rate of 0.2 Gy/s (blue dots) and 2 Gy/s (green dots).

To compare the impact of 1 MeV accelerated electrons and 80 keV X-rays at different dose rates on the decomposition rate of hexanol, the degradation efficiency of 1-hexanol ε was calculated as follows:

(1) $$\varepsilon \left(\%\right)=\frac{C_{H}\left(0\right)-C_{H}\left(D\right)}{C_{H}\left(0\right)}\times 100,$$

where D (Gy) is the irradiation dose, C_H_(0) and C_H_(D) (mg/L) are the initial concentration of 1-hexanol and the concentration of 1-hexanol after irradiation with the dose D, respectively. Figure 7 compares the degradation efficiency of 1-hexanol irradiated with accelerated electrons and X-rays at different dose rates.

Figure 7 shows that when 1-hexanol is irradiated with accelerated electrons, high degradation efficiency (>70%) is achieved at the dose of 1200 Gy. At the same time, there is practically no difference (*p*-value = 0.49) between the dose rates of 1 and 10 Gy/s. In the case of X-ray irradiation at the dose rate of 2 Gy/s, the degradation efficiency of 1-hexanol is more than 70% only at 4 kGy, which is three times less efficient than that observed for accelerated electrons. It should be noted that there is a marginal effect (*p*-value = 0.10) of dose rate on hexanol degradation.

The fact that practically no difference between the hexanol degradation efficiency in the solution irradiated at the dose rates ranging from 0.2 Gy/s to 10 Gy/s was observed is consistent with the observations of other authors stating that the radiation–chemical yield of reactive oxygen species in 4% albumin solutions under the action of e-beam irradiation at the dose rate ranging from 1.4 Gy/s to 8.7 Gy/s practically does not depend on the dose rate and is within the measurement error [59]. On the other hand, the study of the effect of dose rate on the release of radicals e^−^_aq_, H_2_O_2_, etc. in water irradiated 230 MeV protons shows a decrease in G-value with an increase in the dose rate from 0.1 to 50 Gy/s [60]. On the contrary, another recent study of the effect of gamma irradiation dose rate on ethanol, methanol, and NaBr solutions shows an increase in the radiation yield of H_2_O_2_ with an increase in the dose rate from 3.2 Gy/s to 0.2 Gy/s [42]. Therefore, the effect of the dose rate on degradation of chemical compounds in water solutions as a result of irradiation depends not only on the type of irradiation but also on the extent to which the dose rates are different.

To quantify the effect of electrons and X-rays on hexanol degradation, the experimental dependencies of degradation efficiency on the dose can be approximated by the following function:

(2) $$\varepsilon \left(\%\right)=100\%(1-e^{-kD}),$$

where *k* is the hexanol decomposition rate, indicating how much hexanol is decomposed per unit of the absorbed dose. The G-values are calculated after irradiation of the hexanol solution with the dose of 1200 Gy and the decomposition rate *k* for e-beam and X-rays at different dose rates are shown in Table 1.

As it can be seen, irradiation of 1-hexanol with accelerated electrons showed a higher radiation–chemical yield compared to X-ray irradiation: G_1200Gy_ = 2.81–2.91 molecules/100 eV (e-beam) and G_1200Gy_ = 0.84–1.54 molecules/100 eV (X-rays). The comparison of radiation–chemical yield of VOCs during 1 MeV electron beam irradiation and 80 keV photons at the close dose rates shows that the degradation efficiency of 1-hexanol is higher for electrons than that for X-ray irradiation which can be explained by higher radiation–chemical yield for some radicals, such as OH●, e^−^_aq_ and H_3_O^+^ as well as the different nature of the interaction of electrons and X-rays with water, different dose distribution, and linear energy transfer distribution. These results generally align with G-values for aerated Fricke dosimetry solution showing that the G-value 15.5 ± 0.2 ions/100 eV, which was measured in the dosimetry solution irradiated with electrons, is higher than the G-value 14.7 ± 0.3 ions/100 eV for photons [61,62].

The analysis of studies on the radiation–chemical yield of aliphatic alcohols and their derivatives shows that G values vary from 0.1 to 3.2 molecules/100 eV, depending on the type of irradiation, dose rate initial concentrations of alcohols in aqueous solutions, and aeration with oxygen [63,64,65,66,67]. For instance, in [67], the authors irradiated ethanol in the water solution and revealed that radiation–chemical yield (G) of acetaldehyde, which is the main decomposition product of ethanol, increased from 1.2 to 3.2 molecules/100 eV with the increase in the initial ethanol concentration from 1% to 95%, which generally coincides with the findings presented in this article.

### 2.5. 1-Hexanol Decomposition Mechanisms Triggered by Irradiation

Since irradiation transfers energy in discrete portions, volatile molecules break down into smaller structures as they rearrange as a result of the breaking of chemical bonds. The extent of molecular transformations depends on the bonding energy and molecular structure of the compound as well as the physical parameters of irradiation, such as particle flux density, energy spectrum, dose uniformity, the linear energy transfer values, as well as the spatial distribution of water radiolysis products. The difference in irradiation parameters between e-beam and X-rays, including energy spectrum, explains why the same VOCs identified in 1-hexanol solution behave differently when the same irradiation dose is applied.

During a 1 MeV electron beam irradiation primary electrons and secondary electrons ejected from the matter can produce direct ionization of the matter. Direct ionization of 1-hexanol molecules, further referred to as the Ionizing Reaction (IR), caused by primary and secondary electrons when irradiated with accelerated electrons (e^−^), initiate the breaking of chemical bonds. 1 MeV accelerated electrons can also trigger electron–photon cascades causing secondary electrons to occur and contribute to direct ionization of the matter. Similarly, when exposed to X-rays (γ), secondary electrons occurring as a result of the Compton effect break chemical bonds causing IR. Secondary electrons can cause bremsstrahlung photons to occur; thus, triggering electron–photon cascades. All secondary electrons contribute to direct ionization of the matter. The presence of oxygen causes additional oxidation of 1-hexanol molecules, further referred to as the Oxidation Reaction (OR).

Another mechanism of 1-hexanol degradation in water solutions is indirect action of irradiation occurring in water and involving radiolysis products. The intensity of indirect action of irradiation going through water radiolysis products, such as hydroxyl radical •OH, hydrated e^−^_aq_, and hydrogen atom H• with 1-hexanol molecules or its degradation products, known as Radical Reaction (RR), is determined by the number of free radicals as well as the diffusion coefficients of radicals and the molecular structure of the compounds. Radical reactions play a major role in the transformations of molecules in aqueous environments [68]. Both IR with/without OR and RR lead to the formation of new low-molecular-weight compounds, which are probabilistic in nature and can be described using logistic functions [5].

Since hexanol undergoes different radiation-induced chemical reactions resulting in various decomposition products, Density Functional Theory (DFT) involving thermodynamic calculations of radical reactions, oxidation reactions, and direct ionization was used to explore the most probable mechanisms behind the formation of 1-hexanol decomposition products. To find the degradation products which are most likely to occur in the 1-hexanol solution as a result of irradiation, we have examined the thermodynamic values of the bond energies E (kcal/mol) and the bond energies involving the energies of molecular vibrational motions E_0_ (kcal/mol), whose rupture leads to the formation of intermediate and final decomposition products identified in the hexanol solution, as well as the Gibbs energies G (kcal/mol) and the structures of all identified volatile organic compounds. All energies and the molecular structures of the 1-hexanol decomposition products calculated using DFT method are given in Table A5, in Appendix A.

**Figure 8:**
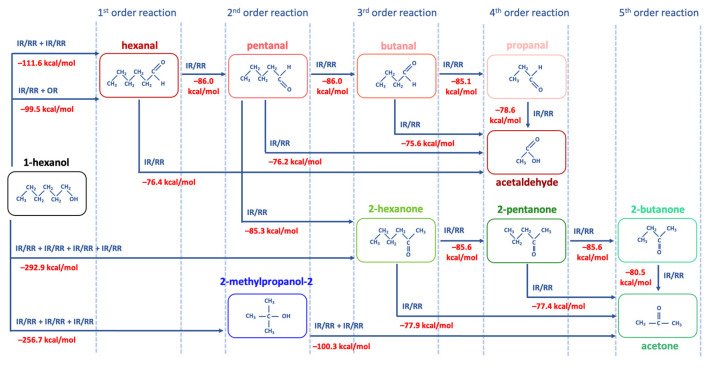
Transformation of 1-hexanol to secondary decomposition products.

**Figure A1:**
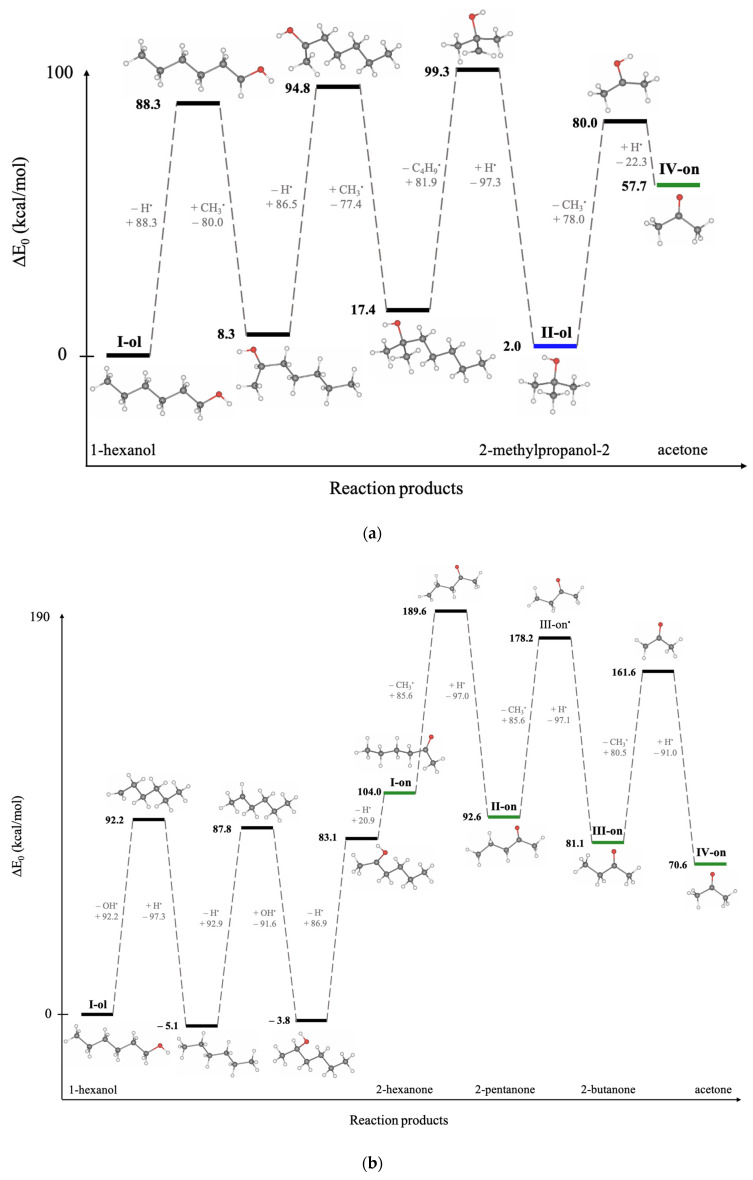

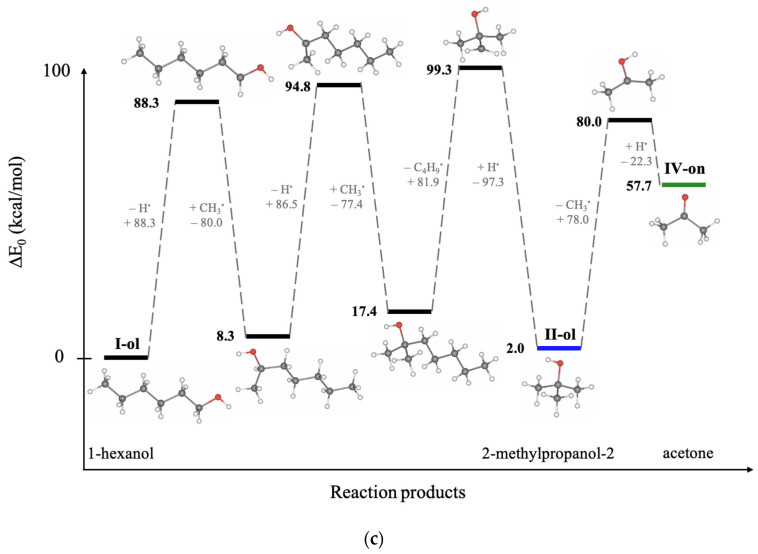
DFT-calculated energies of possible reaction pathways of 1-hexanol after ionizing irradiation, leading to the formation of aldehydes (**a**), ketones (**b**), and alcohols (**c**). The energy of 1-hexanol is taken as the reference (0.0 au).

Figure 8 below illustrates the structures of 1-hexanol secondary decomposition products identified in the 1-hexanol solution and the energy ∆E_0_ used to form secondary decomposition products; ∆E_0_ is the difference between the energy of the identified compound and the one preceding it taking into account all potential intermediate compounds. The detailed DFT calculations of the radiation-induced reactions for the conversion of 1-hexanol into degradation products are shown in Figure A1, in Appendix A.

Since hexanal is the main contributor to the radiation–chemical yield of 1-hexanol decomposition, the other VOCs, whose formation is more complex in nature as they undergo multiple transformations due to direct and indirect ionization, are secondary oxidation products of 1-hexanol.

DFT calculations performed to determine the most probable ways of the 1-hexanol transformations to decomposition products identified in the 1-hexanol solution after irradiation show that the probable transformation of 1-hexanol to hexanal occurs by breaking C–H and C–O bonds involving different ionization types:

1. Direct Ionizing Reaction (IR):

1-Hexanol (I-ol) + e^−^ → Radical 1-Hexanol (I-ol^●^) + H^●^

I-ol^●^ + e^−^ → Hexanal (I-al) + H^●^

2. Indirect Ionizing Reaction—Radical Reaction (RR):

I-ol + R^●^ (H^●^/OH^●^) → I-ol^●^ + H^●^

I-ol^●^ + R^●^ (H^●^/OH^●^) → I-al + H^●^

3. IR/RR and subsequent Oxidation Reaction (OR):

I-ol + e^−^/R^●^ → I-ol^●^ + H^●^

I-ol^●^ + O_2_ → I-al + HO_2_^●^

**Figure 9:**
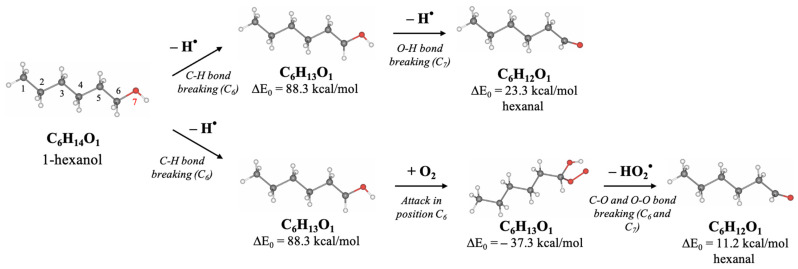
The mechanism behind 1-hexanol transformation into hexanal. Carbon atoms are shown in gray, hydrogen atoms in white, and oxygen atoms in red.

Figure 9 illustrates the proposed mechanism of hexanal formation from 1-hexanol due to irradiation. The initial compound 1-hexanol uses a structural formula where carbon atoms are numbered from 1 to 6. At first, the cleavage of C–H bonds occurs either through direct ionization or radiation-induced hydrogen atom (H•) abstraction. After that, as the concentrations of ions or radicals in the medium increase, further abstraction of a hydrogen atom can occur, leading to the formation of hexanal. An alternative pathway involves a radical reaction with molecular oxygen (O_2_), followed by conversion to hexanal through the formation of a peroxyl radical and a hydroperoxide intermediate.

This mechanism corresponds to the typical process of radiation-induced peroxidation of saturated alcohols, fatty acids, and alkanes in aqueous or oxygen-rich environments. The mechanism includes the initiation of radical reactions, their propagation through chain processes, and the formation of stable oxidation products, particularly aldehydes such as hexanal, which are often used as markers of oxidative degradation [69,70,71].

The energies and the formation pathways of other degradation products of 1-hexanol are presented in Figure A1, in Appendix A.

IR or RR involves the detachment of two hydrogen atoms H^●^ one after the other, whereby ∆E_0_ = 111.6 kcal/mol is expended. In the presence of oxygen O_2_, the IR/RR reactions are followed by the OR, which lowers the energy to ∆E_0_ = 99.5 kcal/mol, increasing the probability of this reaction. Secondary oxidation products of 1-hexanol, however, require more energy due to a greater number of ionization events. Thus, to obtain a 2-hexanone ketone, it is necessary to break four bonds, expending ∆E_0_ = 292.9 kcal/mol, whereas the formation of alcohol 2-methypropanol-2 requires ∆E_0_ = 256.7 kcal/mol.

The transformation pathways outlined in Figure 8 show that each compound formed can be categorized into groups based on the order of their formation. The first group includes reactions of the first kind, which involve the formation of hexanal from 1-hexanol through OR and/or RR/IR. The second group consists of compounds formed by the radical or ionizing reaction of hexanal (e.g., pentanal) or those that have more complex production pathways directly from 1-hexanol (e.g., 2-methylpropanol-2). The VOCs of the third group, such as butanal and 2-hexanone and the fourth and fifth group, such as acetaldehyde, propanal, 2-pentanone, 2-butanone, and acetone can be a daughter product of the first and the second groups or the decomposition product of primary 1-hexanol molecules. The analysis of mechanisms behind irradiation-induced transformations of 1-hexanol reveals that hexanal is a high-probability decomposition marker of 1-hexanol since, unlike other VOCs, hexanal requires only two ionization events, either two direct ionizations or two radicals H^●^/OH^●^, or one direct ionization or a radical reaction combined with oxidation.

### 2.6. Mathematical Model Describing VOCs Transformations Under Irradiation

For quantitative estimation of the radiation–chemical yield of low-molecular-weight compounds and molecule transformations under irradiation, we propose a mathematical model describing the dynamics of the transformation of 1-hexanol into hexanal. Since irradiation of the 1-hexanol solution caused 1-hexanol concentration to decrease while forming other secondary VOCs, the summary concentration of the resultant low-molecular-weight compounds can be expressed as:

(3) $$C_{H}(0)-C_{H}\left(D\right)=\sum C_{VOCs}\left(D\right),$$

where D (Gy) is the irradiation dose, C_H_(0) and C_H_(D) (mg/L) are the initial concentration of 1-hexanol, and the concentration of 1-hexanol after irradiation with the dose D, respectively, ∑C_VOCs_ (mg/L) is the sum of the VOCs concentrations formed as a result of 1-hexanol degradation.

**Figure 10:**
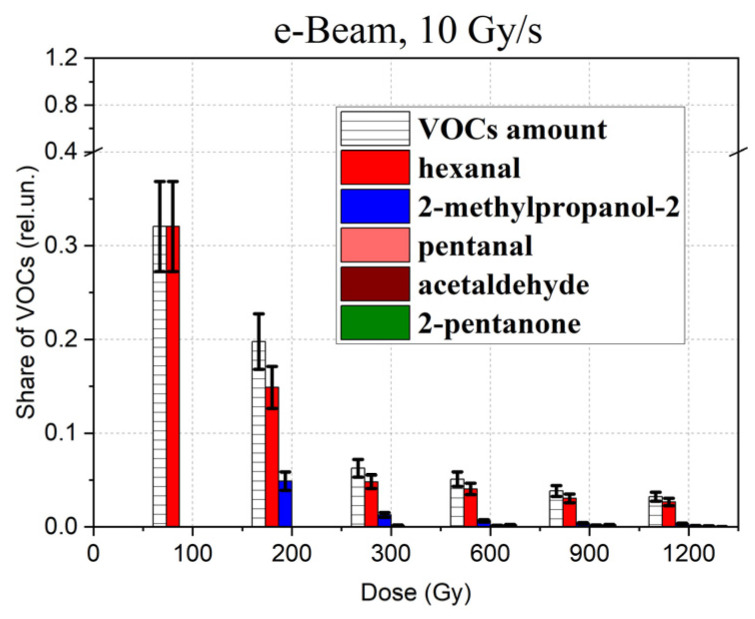
Dose dependencies of the share of 1-hexanol degradation VOCs *A*(D) identified in the 1-hexanol solution irradiated with accelerated electrons at dose rate 10 Gy/s in the total amount of decomposed 1-hexanol molecules.

Since secondary VOCs were found in different concentrations in 1-hexanol solutions irradiated with different doses, the share of VOCs appearing as a result of 1-hexanol degradation in the total amount of 1-hexanol molecules was estimated as shown in Figure 10 using the following formula:

(4) $$A\left(D\right)=\frac{C_{i}(D)}{C_{H}(0)-C_{H}\left(D\right)},$$

where *A*(D) is the share of 1-hexanol degradation VOCs identified in the 1-hexanol irradiated with the dose D; C_i_(D) (mg/L) are the concentrations of VOCs *i* = 1 (hexanal), *i* = 2 (2-methylpropanol-2), *i* = 3 (pentanal), etc., in the 1-hexanol irradiated with dose D.

As can be seen from Figure 10, the share of VOCs decreases exponentially with an increase in the irradiation dose, which can be expressed using the following formula:

(5) $$A\left(D\right)={A_{0}e}^{-BD},$$

where *A*_0_ is the initial relative concentration of 1-hexanol molecules in the non-irradiated suspension, *B* is the rate at which the share of 1-hexanol degradation VOCs decrease with a higher dose.

The fact that the share of total VOCs identified as a result of 1-hexanol degradation in the total amount of 1-hexanol molecules is under 100% suggests that some VOCs, such as oxygen, water, carbon dioxide, formaldehyde, etc., could not be identified since their molecular weight is beyond the range of m/z values 33–350 which can be registered using the given scan mode:

(6) $$C_{H}(0)-C_{H}\left(D\right)=\sum C_{VOCs}\left(D\right)+\sum C_{VOCs}^{\prime }\left(D\right),$$

where ∑C_VOCs_ and ∑C′_VOCs_ are the sum of the concentrations of the identified and non-identified VOCs, respectively.

The exponential decrease in the concentration of 1-hexanol *C*_H_ with an increase in the dose (Figure 4a–d) can be described by the following differential Equation (7):

(7) $$\left\{\begin{array}{c} \frac{dC_{H}}{dD}=-k_{H}C_{H}\\ C_{H}\left(0\right)=C_{H}^{0} \end{array}\right.,$$

where *k*_H_ (Gy^−1^) is the decomposition rate of 1-hexanol molecules and $C_{H}^{0}$ = 50 mg/L is the 1-hexanol concentration in the non-irradiated suspension. The solution of Equation (7) shows that *k*_H_ reflects the dose at which the concentration of 1-hexanol decreases by *e* times:

(8) $$C_{H}\left(D\right)={C_{H}^{0}e}^{-k_{H}D}.$$

The non-linear dependency of hexanal concentration on the irradiation dose after X-ray irradiation with a wide dose range (Figure 4d) is a clear sign of two competing processes—the accumulation of hexanal as a result of 1-hexanol decomposition and the decomposition of hexanal as a result of irradiation. The differential equation which describes a change in the hexanal concentration with an increase in the irradiation dose can be expressed as follows:

(9) $$\left\{\begin{array}{c} \frac{dC_{h}}{dD}=-k_{h}C_{h}+A_{Hh}(D)\cdot C_{H}(D)\\ C_{h}\left(0\right)=0 \end{array}\right.,$$

where *C*_h_ (mg/L) is the concentration of hexanal identified in the irradiated 1-hexanol suspension, *k*_h_ (Gy^−1^) is the decomposition rate of hexanal, *A*_Hh_(D) is the share of 1-hexanol molecules which transform to hexanal in the total amount of 1-hexanol molecules and *C*_h_(0) is the concentration of hexanal in the non-irradiated 1-hexanol solution.

Taking into account that the share of hexanal *A*_Hh_(D) formed due to 1-hexanol degradation in the total amount of decomposed 1-hexanol molecules decreased exponentially with an increase in the irradiation dose Equation (5), the following dose dependency of the hexanal concentration was obtained from Equation (9):

(10) $$C_{h}\left(D\right)=\frac{A{\cdot C}_{H}^{0}\cdot k_{h}}{{B+k}_{H}-k_{h}}\cdot \left(e^{-k_{h}D}-e^{-BD}e^{-k_{H}D}\right)+C_{h}^{0}{\cdot e}^{-k_{h}D}.$$

**Figure 11:**
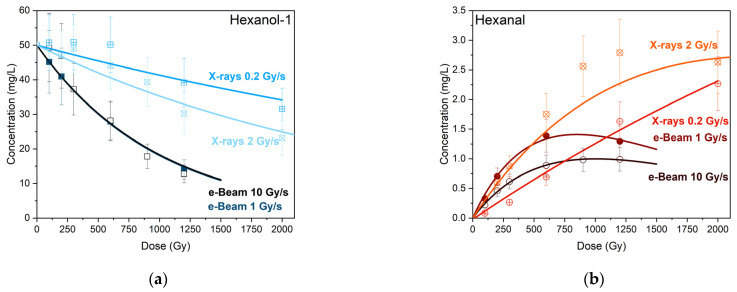
Experimental dependencies (symbols) and calculated dependencies (lines) of the concentrations of 1-hexanol (**a**) and hexanal (**b**) on the irradiation dose up to 2000 Gy when irradiated with accelerated electrons and X-rays.

Figure 11a,b shows the experimental dependencies and dependencies of the concentrations of 1-hexanol and hexanal in the 1-hexanol solutions irradiated with e-beam and X-rays at different dose rates calculated using Equations (8) and (10).

As can be seen from Figure 11, accelerated electrons led to a more considerable degradation of 1-hexanol and its main degradation product hexanal compared to X-ray irradiation, which can be attributed to a higher ionization density throughout the whole volume of 1-hexanol solution and a higher dose rate during e-beam irradiation.

Table 2 shows the values of the coefficients $C_{H}^{0}$, *k*_H_, *k*_h_, *A*, and *B* calculated using Equations (8) and (10) for 1-hexanol and hexanal identified in the 1-hexanol solutions irradiated with accelerated electrons and X-rays at different dose rates. The order of magnitude differences in the decomposition rate of both compounds under different types of irradiations indicate a more significant influence of electron irradiation on volatile compounds compared to X-ray irradiation. The fact that the decomposition rates of 1-hexanol and hexanal are similar in order of magnitude is a clear sign that the volatile organic compounds from different classes with similar chemical structure and molecular weight have a similar decomposition mechanism as a result of irradiation.

The formation of the other VOCs, consisting of multiple transformations of 1-hexanol secondary decomposition products with concentration C_j_ can be expressed as follows:

(11) $$\left\{\begin{array}{c} \begin{array}{c} \begin{array}{c} \frac{dC_{j}}{dD}=-k_{j}C_{j}\\ \frac{dC_{i}}{dD}=-k_{i}C_{i}+A_{ij}(D)\cdot C_{j}(D)\\ A_{ij}(D)=\left\{\begin{array}{c} A_{ij}(D),\;D>D_{0}\\ 0,\;D\leq D_{0} \end{array}\right. \end{array}\\ C_{j}\left(0\right)=C_{j}^{0} \end{array}\\ C_{i}\left(0\right)=0 \end{array}\right..$$

In this formula C_j_ (mg/L) is the concentration of compound *j*, C_i_ (mg/L) is the concentration of compound *i* formed from compound *j*; *k*_j_ and *k*_i_ (Gy^−1^) are the decomposition rates of compound *i* and *j*, respectively, and *A_ij_*(D) is the share of transition of compound *j* to compound *i* in the total amount of compound *j* molecules taking into account the threshold dose D_T_ at which compound *i* was identified. The solution to Equation (11) can be represented as:

(12) $$\left\{\begin{array}{c} C_{j}\left(D\right)={C_{j}^{0}e}^{-k_{j}D}\\ C_{i}\left(D\right)=\left\{\begin{array}{c} e^{-k_{i}D}\cdot \int_{D_{0}}^{D}{C_{j}^{0}e}^{-\left(k_{j}-k_{i}\right)x}A_{ij}\left(x\right)dx,D>D_{T}\\ 0,D\leq D_{T} \end{array}\right. \end{array}\right..$$

Considering that the compound *i* can not only occur as a result of 1-hexanol decomposition but can also be a daughter product of 1-hexanol decomposition VOCs and solving the equation requires a great number of input parameters which are hard to identify, further research of the behavior of these compounds is not deemed feasible. Hexanal dose-dependent behavior, however, has proved to be easy to trace and estimate which makes hexanal a quantitative marker of irradiation dose-induced decomposition of 1-hexanol. The application of Equation (10) approximating the experimental dose dependency for hexanal allows to determine the quantitative parameters of dose-dependent 1-hexanol. Knowing the initial concentration of 1-hexanol, it is possible to determine the extent at which 1-hexanol molecules decompose after irradiation with a certain dose.

**Figure 12:**
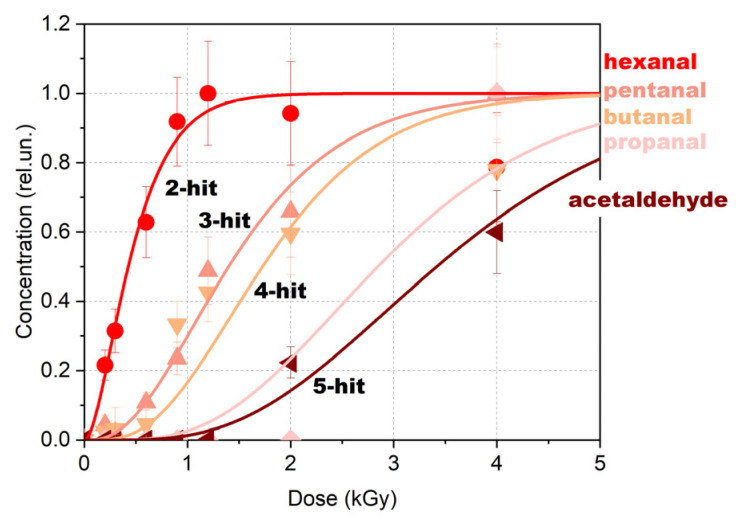
Multi-hit dose–response model for the formation of 1-hexanol decomposition products as a result of X-ray irradiation. Experimental data are shown as dots, calculated dependencies are shown as lines.

Having analyzed the results of DFT calculations, we elaborated an alternative approach based on radiobiological target theory which is believed to be a more efficient for describing the dose-dependent behavior of the secondary 1-hexanol decomposition products. DFT calculations show that the formation of decomposition products requires two to five ionization events depending on the type of decomposition product ensuing the appearance of certain compounds which can be identified at the dose exceeding the threshold dose D_T_. The dose behavior of these compounds can thus be described using a multi-hit dose–response model [72,73,74]. According to this model, the probability of the appearance of a certain compound can be estimated as follows:

(13) $$P\left(D\right)=1-\sum_{k=0}^{k=n-1}\frac{{\left(\lambda D\right)}^{k}e^{-\lambda D}}{k!},$$

where *n* is the number of ionizing events, further referred to as hits, triggering the formation of a certain compound, whereas λ is the formation rate of a certain compound per unit dose. Using this approach, dose dependencies for the 1-hexanol decomposition products can be represented as shown in Figure 12, which shows the number of hits required for the formation of a certain compound as per DFT calculations.

As can be seen from Figure 12, an increase in the number of hits causes a transition from an exponential dose dependency to a sigmoidal one that highlights the threshold dose at which a certain compound is identified in the 1-hexanol solution. For example, while hexanal formation requires two hits leading to an exponential dependency of the hexanal concentration on the irradiation dose, acetaldehyde requires five hits which determines a sigmoidal dose dependency. The multi-hit dose response-based approach described in this section has proved the validity of the mathematical model provided below.

## 3. Materials and Methods

### 3.1. Object of the Study

The research object is a solution of the volatile organic compound 1-hexanol alcohol for gas chromatography (CAS Number: 111-27-3; 1-Hexanol reagent grade, 98%. Sigma-Aldrich: St. Louis, MO, USA, 2024), diluted in a 0.9% (*w*/*v*) saline solution to an initial concentration of 50 mg/L, further referred to as “the 1-hexanol solution”. An aliquot of the 0.5 mL solution was placed into ∅ 9 mm, 3.9 cm long Eppendorf-type microcentrifuge polypropylene tubes with a volume of 2 mL. Out of the total of 162 tubes with the 1-hexanol solution, 60 tubes were irradiated with accelerated electrons, and 78 tubes were irradiated with bremsstrahlung photons, in two iterations. The remaining twenty-four samples were used as non-irradiated control samples.

### 3.2. Electron Beam and X-Ray Irradiation

**Figure 13:**
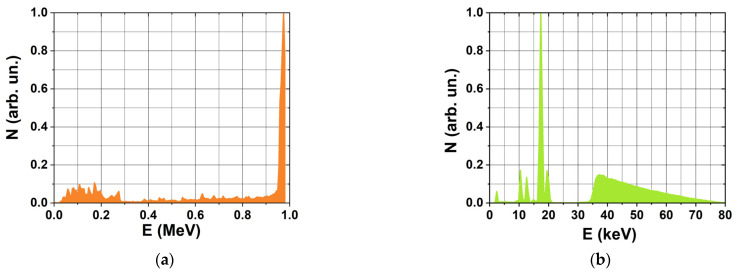
1-Hexanol suspension irradiation method involving UELR-1-25-T-001 electron accelerated with energy spectrum (**a**) and the X-ray apparatus RAP 100-10 with energy spectrum (**b**).

The 1-hexanol suspension samples were irradiated with 1 MeV accelerated electrons generated by a continuous electron linear accelerator UELR-1-25-T-001 (Skobeltsyn Scientific Research Institute of Nuclear Physics, Moscow, Russia) with an average beam power of 25 kW. The spectrum of the electron beam is shown in Figure 13a. For each irradiation session, six samples were placed on a 35 cm × 5.2 cm duralumin plate located 12 cm from the beam output, according to the irradiation method shown in Figure 1. During electron beam irradiation with the dose rate of 1 G/s four irradiation doses were applied to the total of twenty-four samples. For the dose rate of 10 G/s, thirty-six samples were irradiated with six doses. The total number of non-irradiated samples was twelve. The average beam current of 0.2 µA ensured that the dose rate absorbed by the 1-hexanol suspension samples was 1 Gy/s and a beam current of 2.0 µA allowed reaching the dose rate of 10 Gy/s.

The 1-hexanol suspension samples were irradiated with bremsstrahlung photons generated by the X-ray apparatus RAP 100-10 with a 1BPV 23-100 X-ray tube (Burnazyan FMBC, Moscow, Russia) and a molybdenum anode set to perform at U_A_ = 80 kV. The X-ray energy spectrum is shown in Figure 13b. Three 1-hexanol suspension samples were placed at a distance of 12 cm from the beryllium window and irradiated simultaneously with X-rays (Figure 1). Two independent irradiation series were implemented for each irradiation dose. During X-ray irradiation with the dose rate of 0.2 G/s, five doses were applied to thirty samples. For the dose rate of 2 G/s, forty-eight samples were irradiated with eight doses. Twelve samples were not irradiated and kept as controls. The average tube beam of 0.8 mA ensured that the dose rate absorbed by the 1-hexanol suspension samples was 0.2 Gy/s and the tube beam 8.0 mA allowed to reach the dose rate of 2 Gy/s.

### 3.3. Computer Simulation

The GEANT4 toolkit based on the Monte–Carlo method was used to calculate dose and LET distribution in vials, irradiated with accelerated electrons and X-rays.

To determine the absorbed dose and LET distribution in the vials irradiated with 1 MeV e-beam and 80 keV X-rays, a single vial was simulated as a 9 mm × 39 mm hollow cylinder with polypropylene walls (1 mm thick), partially filled with water.

Each simulation of the e-beam irradiation method was performed using 10^8^ electrons with the energy spectrum shown in Figure 13a. Each simulation of X-ray irradiation was performed using 10^9^ photons with the energy spectrum shown in Figure 13b. In total, 2 computer simulations were performed.

To calculate depth-dose distributions and LET values in vials during e-beam irradiation and X-ray irradiation, the water phantom was divided into 20 × 20 × 20 cells. The energy dE absorbed by each cell was estimated during computer simulation, and then the dose D absorbed by each cell was determined using the following formula:

(14) $$D_{cell}=\frac{{dE}_{cell}}{{dm}_{cell}},$$

where dm_cell_ is the mass of the cell.

The average value of LET ($\bar{L_{D}}$) over the absorbed energy was calculated by the formula:

(15) $$\bar{L_{D}}=\sum_{i=1}^{N}\frac{∆E_{i}}{∆l_{i}}w_{i},$$

where ∆E*_i_* is the energy released by the *i*-th electron in the sample cell, ∆*l_i_* is the length of the track of the *i*-th electron among all N electrons in the sample cell, and *w*i is the weight factor determined by the formula:

(16) $$w_{i}=\frac{{∆l}_{i}}{\sum_{j=1}^{N}{∆l}_{j}}.$$

Geant4-DNA extends the Geant4 Monte–Carlo simulation toolkit for radiation biophysics and radiobiology. Geant4-DNA conducts simulations in stages: the physical-chemical stage (the thermalization process of electrons and events that are occurring in ionized and excited water molecules) and the chemical stage (the chemical species diffuse through the medium and interact with each other). Our study uses an online version of Geant4-DNA (version 11.3.2) to calculate G-values for electrons and photons. Example “chem6” is used as a basis for the calculations. The physics settings are as follows: the G4EmDNAPhysics_option2 physics constructor and G4EmDNAChemistry_option3 chemistry constructor. The physical-chemical stage lasts up to 1 picosecond and the chemical stage lasts from 1 picosecond up to 1 microsecond.

### 3.4. Dosimetry Control

Ferrous sulfate (Fricke) dosimeter was used to estimate the dose absorbed by the 1-hexanol suspension samples irradiated with e-beam and X-ray irradiation. The Fricke solution containing 0.4 M sulfuric acid, 0.2 mM ferric ammonium sulfate, and 0.26 mM potassium chloride was prepared using ultrapure water and thoroughly mixed to saturate the solution with air according to the methodology described in [75].

The dose absorbed by the Fricke solution was calculated using the following formula:

(17) $$D=\frac{k∆S\left({Fe}^{3+}\right)}{\rho l\varepsilon G\left({Fe}^{3+}\right)},$$

where ε = 2160 L⋅mol^−1^ × cm^−1^ is the molar extinction coefficient of Fe^3+^ ions, *l* = 1 cm is the optical path length, *k* = 9.65 × 10^6^ is a dimensionless coefficient, and *ρ* = 1.024 g⋅cm^−3^ is the density of the dosimetry solution. For electrons, the radiation–chemical yield G(Fe^3+^) is 15.6 ions/100 eV and for photons it is 14.4 ions/100 eV. Analysis of the literature shows that no significant effect [76] of dose rate up to 50 Gy/s on the radiation–chemical yield in the Fricke solution is found. It should be noted that a significant decrease in the G-values is only registered when the dose exceeds 1000 Gy/s [77].

The irradiation method for the Fricke dosimeter fully corresponded to the electron beam irradiation and X-ray irradiation method for 1-hexanol suspension in 2.0 mL polypropylene Eppendorf tubes. The absorbed dose was determined from the measured optical density of the FeSO_4_ solution in the range of 40–400 Gy, corresponding to the working range of the diluted Fricke dosimetry solution. For X-rays, the exposure time varied from 300 to 1800 s with a 300 s increment with six irradiation sessions in total, while for electrons, the exposure time varied from 60 to 360 s with a 60 s increment with six irradiation sessions in total. The optical density of the Fricke dosimeter solution was measured at a wavelength of λ = 304 nm on a UV-3000 spectrophotometer (TM ECOVIEW, St. Petersburg, Russia) to estimate the dose rate absorbed by the 1-hexanol suspension samples [78].

**Figure 14:**
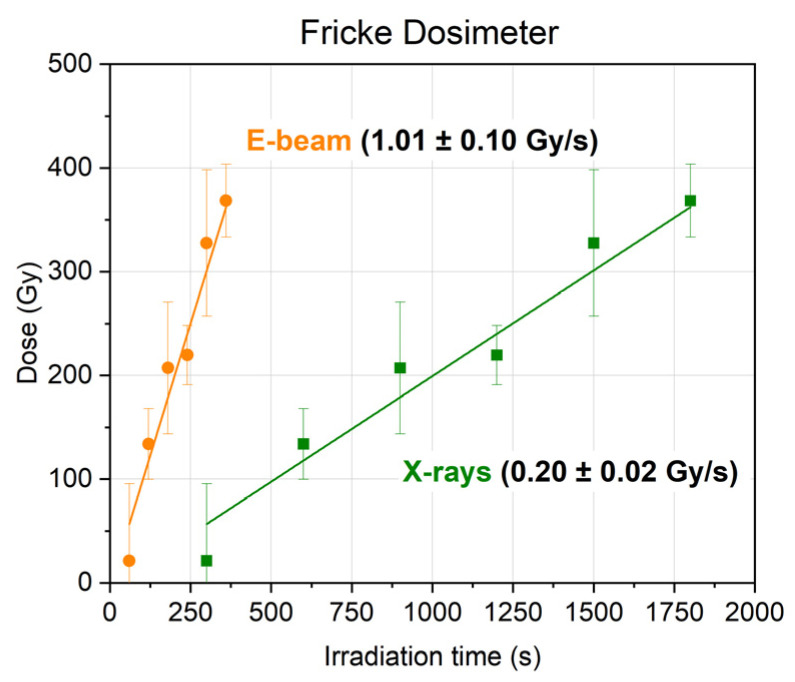
The dependency of the dose absorbed by the Fricke dosimeter upon irradiation with accelerated electrons (orange dots) and X-ray photons (green dots) on the irradiation time.

Figure 14 shows the dependences of the dose absorbed by the ferro sulfate dosimeter on the irradiation time. The dose rates were determined as the slope coefficient of the linear approximation of experimental data.

1-Hexanol suspension samples were irradiated with e-beam X-rays with the doses ranging from 100 Gy to 1200 Gy at the dose rates of (1.0 ± 0.1) Gy/s and (10 ± 1) Gy/s. The 1-hexanol suspension samples were exposed to X-ray irradiation with the doses ranging from 100 Gy to 2000 Gy at the dose rate of (0.20 ± 0.02) Gy/s and the doses ranging from 200 Gy to 8000 Gy at the dose rate of (2.0 ± 0.2) Gy/s. Since the density of Fricke’s solution is close to the density of an aqueous solution of hexanol, it can be assumed that the dose rates absorbed by the dosimetry solution coincide with the dose rates absorbed by the experimental alcohol samples.

### 3.5. GC/MS Analysis

After being irradiated, 3.0 mL of 1-hexanol suspension irradiated with the same dose was put into two glass vials, 20 mL each (Shimadzu, Kyoto, Japan), tightly sealed and subjected to GC/MS analysis. The 1-hexanol suspension samples underwent thermal desorption at 95 °C for 20 min, after which 1.5 mL of the vapor phase was injected into a Shimadzu GC/MS-QP2010 Ultra (Shimadzu, Kyoto, Japan) chromatograph equipped with an HT200H Headspace Autosampler (NTA, Avola, Italy) for VOC separation.

The VOCs were separated using a 60 m × 0.32 mm × 1.8 µm VF-624 capillary column (Agilent, Santa Clara, CA, USA). The temperature program for component separation was as follows: the initial temperature was set at 40 °C, followed by an isothermal period of 5 min, then a ramp to 220 °C at a rate of 6 °C/min, and a final isothermal period at the end temperature for 5 min. Helium was used as the carrier gas at a constant flow rate of 1.5 mL/min through the column. The temperature of the evaporator, interface, quadrupole, and ion-source was maintained at 250, 200, 200, and 230 °C, respectively. Electron ionization mass spectra were taken at 70 eV. Chromatograms were recorded in the all-ion scan mode for m/z values ranging from 33 to 350 with 3.3 scans/s.

### 3.6. Analysis of Volatile Compounds

The VOCs were identified by comparing their mass spectra with the spectra from the NIST/EPA/NIH Mass Spectral Library 2008 (NIST 08) using GC/MS solution version 2.70 software (Shimazu, Kyoto, Japan). The concentrations of volatile compounds (mg/L) were calculated using the calibration plot method obtained by chromatographic analysis of standard samples of volatile compounds at different concentrations and peak areas on the chromatogram.

The study uses the following VOC standard samples: Acetaldehyde (99%, 75-07-0, PanReac, Barcelona, Spain), Propanal (98% (GH), 123-38-6, Apollo Scientific, Manchester, UK), Acetone (analytical standard, 67-64-1, Sigma Aldrich, St. Louis, MO, USA), 2-methylpropanol (99+%, for spectrophotometry, 78-83-11, Acros Organics, Geel, Belgium), Butanal, 3-methyl (analytical standard, 290-86-3, Sigma Aldrich, St. Louis, MO, USA), 2-Butanone (For spectrophotometry, 78-93-3, TCI, Tokyo, Japan), 2-Pentanone (analytical standard, 107-87-9, Sigma Aldrich, St. Louis, MO, USA), Pentanal (analytical standard, 110-62-3, Sigma Aldrich, St. Louis, MO, USA), 2-Hexanone (98% (GH), 591-78-6, Sigma Aldrich, St. Louis, MO, USA), Hexanal (analytical standard, 66-25-1, Sigma Aldrich, St. Louis, MO, USA), and 1-Hexanol (standard for GC, 111-27-3, Sigma Aldrich, St. Louis, MO, USA).

To prepare the mix solution of all VOCs, 50 mL of 0.9% (*w*/*v*) saline solution was placed in a 200 mL volumetric flask, and 15–25 mg of individual standard solutions of VOCs were added. The contents of the flask were brought up to the mark with physiological saline and mixed. Then, the resulting solution was placed in an ultrasonic bath for 10 min to enhance the dissolution and homogenization of the VOC mixture.

The relationship between the peak area on the chromatogram and the VOC concentration in the calibration solution was the basis for the calibration curve for each VOC. The RSD values (*n* = 6, *p* = 0.95) for repeated measurements of all VOCs were obtained by the same operator using the instrument within one day and varied from 4% to 15.5%. The metrological characteristics for the determination of VOCs are presented in Table 3.

### 3.7. Statistical Analysis

Statistical and analytical processing of the VOC concentrations was performed using Microsoft Excel v16.0 (Microsoft, Redmond, WA, USA) and OriginPro 2018 SR1 b.9.5.1.195 (Origin Lab Corporation, Northampton, MA, USA). A two-way ANOVA and a three-way ANOVA test was used to compare the radiation–chemical yield of VOCs after irradiation with accelerated electrons and X-rays.

### 3.8. Density Functional Theory Calculations

Calculations of the molecular geometry were performed using the density functional method with the PBE functional [79]. Full electronic basic sets L1 were used, L1 stands for double set size. The number of abbreviated and primitive functions used in L1 is {2,1}/{6,2} for H, {3,2,1}/{10,7,3} for C and O, respectively [80]. Stationary points on the potential energy surface were identified by analyzing the Hessians. The thermodynamic functions, such as bond energy involving the energy of molecular vibrational motions and Gibbs free energy at 298.15 K were calculated using the approximation of a limited rotator and a harmonic oscillator. All calculations were performed on a personal computer using the PRIRODA04 software package [81].

## 4. Conclusions

The study provides evidence that among volatile organic compounds identified in the hexanol solution after e-beam and X-ray irradiation, hexanal can be used as a reliable quantitative marker of hexanol decomposition since its concentration is highly dose-dependent. The density functional theory calculations performed following GC-MS analysis of hexanol solution after irradiation revealed that the transformation to hexanal is a high-probability occurrence for 1-hexanol degradation in comparison with other VOCs, which are decomposition products of 1-hexanol, since they occur as a result of more complex mechanisms. The chaotic nature of dose dependency of secondary VOCs concentrations and the complexity of VOCs formation proved by the DFT calculations and described using a multi-hit dose–response model impedes their effective use as quantitative markers of 1-hexanol decomposition since the mathematical modeling of dose dependencies of secondary VOCs requires multiple input parameters which cannot be obtained using GC-MS analysis of VOCs after irradiation.

Our research suggests that volatile organic compounds have their own high-probability decomposition products which occur due to irradiation and their concentration can be used as a quantitative criterion for determining the dose at which the concentration of initial compounds reduces down to a level which would testify to a significant degradation of the compound in question. Now that we know the mechanisms of degradation of organic molecules after irradiation, in further research we are going to identify VOCs which can be used as markers of degradation of proteins, fats, and carbohydrates to establish more precise dose limits for a wide range of food products commonly irradiated on an industrial scale, taking into account their individual chemical composition. Using the findings obtained in this study and irradiation markers of proteins, fats, and carbohydrates which will be obtained after irradiation of model solutions and real food products will enable the development of a “fast-track” method for establishing the optimal dose range which would not lead to intensive oxidation of proteins, fats, and carbohydrates in irradiated food products.

## Figures and Tables

**Table 1:** G-values and decomposition rate of the 1-hexanol degradation under irradiation with accelerated electrons and X-ray photons.

	E-Beam, 1 Gy·s^−1^	E-Beam, 10 Gy·s^−1^	X-Rays, 0.2 Gy·s^−1^	X-Rays, 2 Gy·s^−1^
*k*, kGy^−1^	1.02 ± 0.02	1.01 ± 0.09	0.19 ± 0.04	0.35 ± 0.03
*G*_1200Gy_(1-hexanol), mol/100 eV	2.81 ± 0.80	2.91 ± 0.79	0.84 ± 0.09	1.54 ± 0.18

**Table 2:** Approximation coefficients describing the transformations of 1-hexanol and its decomposition products.

Type	Dose Rate, Gy/s	$C_{H}^{0}$, mg/L	*k*_H_, Gy^−1^	*k*_h_, Gy^−1^	*A,* rel.un.	*B*, Gy^−1^	R_corr_
e-Beam	1	50	0.0010 ± 0.0004	0.0005 ± 0.0001	0.056 ± 0.008	0.0008 ± 0.0003	0.99
	10		0.0010 ± 0.0004	0.0012 ± 0.0004	0.089 ± 0.010	0.00020 ± 0.00008	0.99
X-rays	0.2		0.00019 ± 0.00002	0.000020 ± 0.000002	0.15 ± 0.03	0	0.98
	2		0.00035 ± 0.00006	0.00040 ± 0.00008	0.18 ± 0.03	0.00011 ± 0.00004	0.99

**Table 3:** VOC validation parameters.

VOC	R2	MDL, mg/L	MQL, mg/L	Linearity Range, mg/L	RSD, %	Accuracy, %
Acetaldehyde	0.998	0.019	0.062	0.13–137.5	12	93
Propanal	0.998	0.017	0.05	0.05–10	14	82
Acetone	0.997	0.011	0.035	0.0398–79.5	15.5	92
2-methylpropanol-2	0.998	0.025	0.10	0.1–10	8.1	91
Butanal,3-methyl	0.998	0.013	0.05	0.05–5	13	92
2-Butanone	0.999	0.0046	0.014	0.014–116.5	8.7	93
2-Pentanone	0.996	0.0029	0.0099	0.0111–111	5	89
Pentanal	0.998	0.020	0.068	0.111–110.5	4	95
2-Hexanone	0.997	0.0035	0.01	0.01–60	8	94
Hexanal	0.999	0.018	0.061	0.061–116.5	5	103
1-Hexanol	0.995	0.27	0.90	0.915–91.5	6	92

## Data Availability

Data are available within the article, Appendix A, and upon request from the corresponding authors.

## Abbreviations

The following abbreviations are used in this manuscript:

VOC	Volatile organic compound
DFT	Density functional theory
GC-MS	Gas chromatography with mass spectrometry
IR	Ionizing Reaction
OR	Oxidation Reaction
RR	Radical Reaction

## Appendix A

**Table A1:** Content of identified compounds in 1-hexanol after electron irradiation in 1 Gy/s dose rate (mg/L; *n* = 2).

Compound	Mol. Wt.	Ret. Time	Concentration, mg/L				
			0 kGy	0.1 kGy	0.2 kGy	0.6 kGy	1.2 kGy
Acetaldehyde	44.053	4.91	ND	ND	ND	ND	0.043 ± 0.009
2-Pentanone	86.13	15.58	ND	ND	ND	ND	0.011 ± 0.002
Pentanal	86.13	15.94	ND	ND	ND	0.036 ± 0.007	0.051 ± 0.010
Hexanal	86.17	19.86	ND	0.33 ± 0.06	0.70 ± 0.14	1.39 ± 0.27	1.29 ± 0.25
1-Hexanol	102.2	22.44	50.0 ± 9.8	45.2 ± 8.9	41.0 ± 8.1	27.9 ± 5.5	14.0 ± 2.8
Sum			50.0	45.5	41.7	29.3	15.4

ND—Not Detected.

**Table A2:** Content of identified compounds in 1-hexanol after electron irradiation in 10 Gy/s dose rate (mg/L; *n* = 2).

Compound	Mol. Wt.	Ret. Time	Concentration, mg/L						
			0 kGy	0.1 kGy	0.2 kGy	0.3 kGy	0.6 kGy	0.9 kGy	1.2 kGy
Acetaldehyde	44.053	4.91	ND	ND	ND	ND	0.049 ± 0.010	0.074 ± 0.015	0.041 ± 0.008
2-methylpropanol-2	74.12	9.088	ND	ND	0.15 ± 0.03	0.16 ± 0.03	0.14 ± 0.03	0.13 ± 0.02	0.13 ± 0.02
2-Pentanone	86.13	15.58	ND	ND	ND	ND	ND	ND	0.009 ± 0.002
Pentanal	86.13	15.94	ND	ND	ND	0.021 ± 0.004	0.034 ± 0.007	0.056 ± 0.011	0.043 ± 0.009
Hexanal	86.17	19.86	ND	0.22 ± 0.04	0.46 ± 0.09	0.62 ± 0.12	0.89 ± 0.17	0.98 ± 0.19	0.99 ± 0.19
1-Hexanol	102.2	22.44	50.0 ± 9.8	49.3 ± 9.7	46.9 ± 9.2	37.3 ± 7.4	28.2 ± 5.6	17.8 ± 3.5	12.7 ± 2.5
Sum			50.0	49.5	47.5	38.1	29.3	19.0	13.9

ND—Not Detected.

**Table A3:** Content of identified compounds in 1-hexanol after X-ray irradiation in 0.2 Gy/s dose rate (mg/L; *n* = 2).

Compound	Mol. Wt.	Ret. Time	Concentration, mg/L					
			0 kGy	0.1 kGy	0.3 kGy	0.6 kGy	1.2 kGy	2.0 kGy
2-methylpropanol-2	74.12	9.088	ND	0.44 ± 0.08	0.55 ± 0.10	0.50 ± 0.10	0.84 ± 0.17	0.87 ± 0.17
Butanal	72.11	11.49	ND	0.035 ± 0.007	0.023 ± 0.004	0.020 ± 0.004	0.026± 0.005	0.033 ± 0.007
2-Pentanone	86.13	15.58	ND	ND	ND	ND	0.047± 0.009	0.061 ± 0.012
Pentanal	86.13	15.94	ND	0.013 ± 0.002	0.011 ± 0.002	0.026 ± 0.005	0.11 ± 0.02	0.17 ± 0.03
Hexanal	86.17	19.86	ND	0.08 ± 0.01	0.26 ± 0.05	0.69 ± 0.13	1.63 ± 0.32	2.27 ± 0.45
1-Hexanol	102.2	22.44	50.0 ± 9.8	50.7 ± 9.6	50.8 ± 10.0	50.1 ± 9.6	39.2 ± 7.8	31.6 ± 6.3
Sum			50.0	51.3	51.6	51.3	41.9	35.0

ND—Not Detected.

**Table A4:** Content of identified compounds in 1-hexanol after X-ray irradiation in the 2 Gy/s dose rate (mg/L; *n* = 2).

Compound	Mol. Wt.	Ret. Time	Concentration, mg/L								
			0 kGy	0.2 kGy	0.3 kGy	0.6 kGy	0.9 kGy	1.2 kGy	2.0 kGy	4.0 kGy	8.0 kGy
Acetaldehyde	44.053	4.91	ND	ND	ND	ND	ND	ND	0.19 ± 0.04	0.50 ± 0.09	0.84 ± 0.17
Propanal	58.08	7.67	ND	ND	ND	ND	ND	ND	ND	0.018 ± 0.003	0.015 ± 0.003
Acetone	58.08	7.81	ND	ND	ND	ND	ND	ND	ND	0.21 ± 0.04	0.33 ± 0.07
2-methylpropanol-2	74.12	9.088	ND	ND	ND	ND	ND	ND	ND	ND	ND
Butanal	72.11	11.49	ND	0.018 ± 0.003	0.024 ± 0.004	0.034 ± 0.007	0.025 ± 0.005	0.032 ± 0.006	0.045 ± 0.008	0.059 ± 0.012	0.075 ± 0.015
2-Butanone	72.11	11.86	ND	ND	ND	ND	ND	ND	ND	ND	0.07 ± 0.01
2-Pentanone	86.13	15.58	ND	ND	ND	ND	ND	0.022 ± 0.004	0.058 ± 0.012	0.11 ± 0.02	0.15 ± 0.02
Pentanal	86.13	15.94	ND	0.023 ± 0.004	0.011 ± 0.002	0.058 ± 0.012	0.13 ± 0.03	0.26 ± 0.05	0.35 ± 0.07	0.53 ± 0.11	0.20 ± 0.04
2-Hexanone	100.2	19.61	ND	ND	ND	ND	ND	ND	ND	0.012 ± 0.002	0.016 ± 0.003
Hexanal	86.17	19.86	ND	0.60 ± 0.12	0.88 ± 0.17	1.75 ± 0.35	2.56 ± 0.51	2.79 ± 0.56	2.63 ± 0.52	2.19 ± 0.44	0.65 ± 0.13
1-Hexanol	102.2	22.44	50.0 ± 9.8	47.2 ± 9.4	48.9 ± 9.7	44.1 ± 8.8	39.4 ± 7.8	30.2 ± 6.0	23.1 ± 4.5	11.2 ± 2.2	4.0 ± 0.8
Sum			50.0	47.8	49.8	45.9	42.1	33.3	26.4	14.8	6.4

ND—Not Detected.

**Table A5:** Thermodynamic parameters of volatile organic compounds.

VOC	E, au	E_0_, au	G, kcal/mol	Structure
Acetaldehyde	−153.688608	−153.635106	18.3	
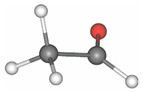
Propanal	−192.957579	−192.875637	34.1	
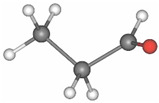
Acetone	−192.967854	−192.886758	34.1	
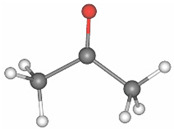
2-methylpropanol-2	−233.433935	−233.302168	64.4	
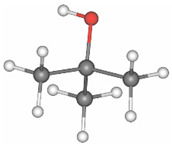
2-Butanone	−232.237310	−232.128222	49.2	
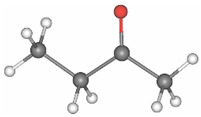
Butanal	−232.225436	−232.115648	50.1	
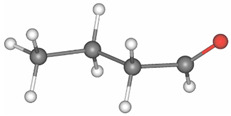
2-Pentanone	−271.504804	−271.367975	65.0	
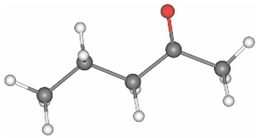
Pentanal	−271.493338	−271.355814	66.0	
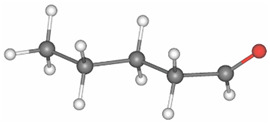
2-Hexanone	−310.772519	−310.608022	80.7	
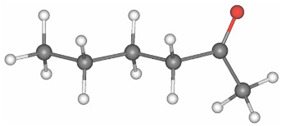
Hexanal	−310.761055	−310.595819	82.0	
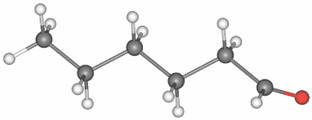
1-Hexanol	−311.959732	−311.771029	96.5	

## References

[1] Chmielewski A.G. (2023). Radiation technologies: The future is today. Radiat. Phys. Chem..

[2] Indiarto R., Pratama A.W., Sari T.I., Theodora H.C. (2020). Food Irradiation Technology: A Review of The Uses and Their Capabilities. Int. J. Eng. Trends Technol..

[3] Chernyaev A.P. (2019). Radiation Technologies. Science. National Economy. Medicine.

[4] Chernyaev A.P., Rozanov V.V., Kozlova E.K., Matveychuk I.V., Bliznyuk U.A., Ipatova V.S. (2025). Radiation Technologies for the Treatment of Biological Objects. Moscow Univ. Phys..

[5] Kozlova E., Bliznyuk U., Chernyaev A., Borshchegovskaya P., Braun A., Ipatova V., Zolotov S., Nikitchenko A., Chulikova N., Malyuga A. (2024). Optimization Function for Determining Optimal Dose Range for Beef and Seed Potato Irradiation. Foods.

[6] Lan B., Zhang S., Tang Z., Cai J., Luo P., Liang S. (2020). Optimization of 60Co γ irradiation process for food. J. Radiat. Res. Radiat. Process..

[7] Domen J.K., Domen S.R. (2001). Studies of Excess Heat and Convection in a Water Calorimeter. J. Res. Natl. Inst. Stand. Technol..

[8] Innovating Radiation Processing of Food with Low Energy Beams from Machine Sources. https://www.iaea.org/projects/crp/d61025.

[9] Baeyens A., Abrantes A., Ahire V., Ainsbury E.A., Baatout S., Baselet B., Botelho M.F., Boterberg T., Chevalier F., Da Pieve F., Baatout S. (2023). Basic Concepts of Radiation Biology. Radiobiology Textbook.

[10] Múčka V., Čuba V. (2024). Radiation sensitivity of biological systems, its modification by chemical modifiers and its quantitative evaluation. J. Radioanal. Nucl. Chem..

[11] Tsutsumi T., Todoriki S., Nei D., Ishii R., Watanabe T., Matsuda R. (2011). Detection of Irradiated Food Using 2-Alkylcyclobutanones as Markers: Verification of the European Committee Standardization Method EN1785 for the Detection of Irradiated Food Containing Lipids. Food Hyg. Saf. Sci. J..

[12] Mazzatenta A., Pokorski M., Di Giulio C. (2021). Volatile organic compounds (VOCs) in exhaled breath as a marker of hypoxia in multiple chemical sensitivity. Physiol. Rep..

[13] Moura P.C., Raposo M., Vassilenko V. (2023). Breath volatile organic compounds (VOCs) as biomarkers for the diagnosis of pathological conditions: A review. Biomed. J..

[14] Le T., Priefer R. (2023). Detection technologies of volatile organic compounds in the breath for cancer diagnoses. Talanta.

[15] Zhou X., Zhou X., Wang C., Zhou H. (2023). Environmental and human health impacts of volatile organic compounds: A perspective review. Chemosphere.

[16] Mangotra A., Singh S.K. (2024). Volatile organic compounds: A threat to the environment and health hazards to living organisms—A review. J. Biotechnol..

[17] Liu X., Jiang H., Xu H., Shang S., Wang D., Bai Y., Zeng F. (2024). Volatile Organic Compounds as Early Detection Indicators of Wheat Infected by *Sitophilus oryzae*. Foods.

[18] Steglińska A., Pielech-Przybylska K., Janas R., Grzesik M., Borowski S., Kręgiel D., Gutarowska B. (2022). Volatile Organic Compounds and Physiological Parameters as Markers of Potato (*Solanum tuberosum* L.) Infection with Phytopathogens. Molecules.

[19] Nieminen T.T., Dalgaard P., Björkroth J. (2016). Volatile organic compounds and Photobacterium phosphoreum associated with spoilage of modified-atmosphere-packaged raw pork. Int. J. Food Microbiol..

[20] Makhlouf L., El Fakhouri K., Kemal S., Aasfar A., Meftah Kadmiri I., El Bouhssini M. (2024). Advances in analytical techniques for assessing volatile organic compounds in pulse crops: A comprehensive review. Front. Hortic..

[21] Bleicher J., Ebner E.E., Bak K.H. (2022). Formation and Analysis of Volatile and Odor Compounds in Meat—A Review. Molecules.

[22] Taiti C., Costa C., Menesatti P., Caparrotta S., Bazihizina N., Azzarello E., Petrucci W.A., Masi E., Giordani E. (2015). Use of volatile organic compounds and physicochemical parameters for monitoring the post-harvest ripening of imported tropical fruits. Eur. Food Res. Technol..

[23] Fischer E., Cayot P., Cachon R., Cayot N. (2023). Effects of ionizing radiation on organic volatile compounds from PEA protein isolate. Heliyon.

[24] Cartoni Mancinelli A., Silletti E., Mattioli S., Dal Bosco A., Sebastiani B., Menchetti L., Koot A., van Ruth S., Castellini C. (2021). Fatty acid profile, oxidative status, and content of volatile organic compounds in raw and cooked meat of different chicken strains. Poult. Sci..

[25] Raza W., Mei X., Wei Z., Ling N., Yuan J., Wang J., Huang Q., Shen Q. (2017). Profiling of soil volatile organic compounds after long-term application of inorganic, organic and organic-inorganic mixed fertilizers and their effect on plant growth. Sci. Total Environ..

[26] Rontani J.F. (2022). Use of Gas Chromatography-Mass Spectrometry Techniques (GC-MS, GC-MS/MS and GC-QTOF) for the Characterization of Photooxidation and Autoxidation Products of Lipids of Autotrophic Organisms in Environmental Samples. Molecules.

[27] Gladilovich V.D., Podolskaya E.P. (2010). Possibilities of applying the GC-MS method (Review). Sci. Instrum..

[28] Dewulf J., Van Langenhove H., Wittmann G. (2002). Analysis of Volatile Organic Compounds Using Gas Chromatography. TrAC.

[29] Xu C.-H., Chen G.-S., Xiong Z.-H., Fan Y.-X., Wang X.-C., Liu Y. (2016). Applications of Solid-Phase Microextraction in Food Analysis. TrAC.

[30] Vas G., Vékey K. (2004). Solid-phase Microextraction: A Powerful Sample Preparation Tool Prior to Mass Spectrometric Analysis. J. Mass Spectrom..

[31] Bliznyuk U., Borshchegovskaya P., Bolotnik T., Ipatova V., Kozlov A., Nikitchenko A., Mezhetova I., Chernyaev A., Rodin I., Kozlova E. (2024). Volatile Compound Markers in Beef Irradiated with Accelerated Electrons. Molecules.

[32] Cao J., Deng L., Zhu X.M., Fan Y., Hu J.-N., Li J., Deng Z.-Y. (2014). Novel approach to evaluate the oxidation state of vegetable oils using characteristic oxidation indicators. J. Agric. Food Chem..

[33] Feng X., Ahn D.U. (2016). Volatile profile, lipid oxidation and protein oxidation of irradiated ready-to-eat cured Turkey meat products. Radiat. Phys. Chem..

[34] Rouseff R.L., Cadwallader K.R. Headspace Analysis of Foods and Flavors: Theory and Practice. Proceedings of the American Chemical Society.

[35] Wang Y., Han J., Wang D., Gao F., Zhang K., Tian J., Jin Y. (2022). Research Update on the Impact of Lactic Acid Bacteria on the Substance Metabolism, Flavor, and Quality Characteristics of Fermented Meat Products. Foods.

[36] Jeong S.M., Kim H.H., Ryu S.H., Kang W.-S., Lee J.-E., Kim S.-R., Lee G.-H., Xu X., Byun E., Ahn D.-H. (2022). Effects of Gamma Irradiation on Inhibition of Urease Activity and Fishy Smell in Mackerel (*Scomber japonicus*) during Refrigerated Storage. J. Microbiol. Biotechnol..

[37] Wen G., Li Z., Choi M.M.F. (2013). Detection of ethanol in food: A new biosensor based on bacteria. J. Food Eng..

[38] Buxton G.V., Greenstock C.L., Helman W.P., Ross A.B. (1988). Critical Review of Rate Constants for Reactions of Hydrated Electrons, Hydrogen Atoms and Hydroxyl Radicals (OH/O− in Aqueous Solution. J. Phys. Chem. Ref. Data.

[39] Johnson D.R., Decker E.A. (2015). The Role of Oxygen in Lipid Oxidation Reactions: A Review. Annu. Rev. Food Sci. Technol..

[40] Abd El H.A.H.M. (2012). Lipid Peroxidation End-Products as a Key of Oxidative Stress: Effect of Antioxidant on Their Production and Transfer of Free Radicals. Lipid Peroxidation.

[41] Ipatova V., Bliznyuk U., Borshchegovskaya P., Chernyaev A., Toropygina M., Kim V., Nikitchenko A., Kozlov A., Yurov D., Beklemishev M. (2025). Assessment of Catalase Inhibition Under E-Beam Irradiation. Int. J. Mol. Sci..

[42] Štefanić I., LaVerne J.A. (2002). Temperature Dependence of the Hydrogen Peroxide Production in the γ-Radiolysis of Water. J. Phys. Chem. A.

[43] de Oliveira Paula M.M., Buchili A.F.M., Rodrigues L.M., Guimarães A.S., Ramos A.d.L.S., Ramos E.M. (2024). Effects of Freezing, Irradiation, and Aging Processes on the Volatile Profile of Nellore Beef. Food Mater. Res..

[44] Begum T., Follett P.A., Hossain F., Christopher L., Salmieri S., Lacroix M. (2020). Microbicidal effectiveness of irradiation from Gamma and X-ray sources at different dose rates against the foodborne illness pathogens *Escherichia coli*, *Salmonella* Typhimurium and *Listeria monocytogenes* in rice. LWT.

[45] Kovács M., Wojnárovits L., Homlok R., Tegze A., Mohácsi-Farkas C., Takács E., Belák Á. (2024). Changes in the behavior of Staphylococcus aureus strains in the presence of oxacillin under the effect of gamma radiation. Environ. Pollut..

[46] Rajkowski K.T., Niebuhr S.E., Dickson J. (2006). Effect of gamma or beta radiation on *Salmonella* DT 104 in ground pork. J. Food Prot..

[47] Marathe S.A., Deshpande R., Khamesra A., Ibrahim G., Jamdar S.N. (2016). Effect of Radiation Processing on Nutritional, Functional, Sensory and Antioxidant Properties of Red Kidney Beans. Radiat. Phys. Chem..

[48] Acquaticci L., Angeloni S., Baldassarri C., Sagratini G., Vittori S., Torregiani E., Petrelli R., Caprioli G. (2024). A new HS-SPME-GC-MS analytical method to identify and quantify compounds responsible for changes in the volatile profile in five types of meat products during aerobic storage at 4 °C. Food Res. Int..

[49] Porrello A., Orecchio S., Maggio A. (2024). Matrix-matched quantification of volatile organic compounds (VOCs) in gluten free flours and bakery products. Food Chem. X.

[50] Liu M., Ji H., Jiang Q., Liu T., Cao H., Zhang Z. (2024). Effects of full shading of clusters from véraison to ripeness on fruit quality and volatile compounds in Cabernet Sauvignon grapes. Food Chem. X.

[51] Oluk A.C. (2022). Effect of production variations on the composition, textural and microstructural properties, and volatile compounds of Turkish white cheese during ripening. LWT.

[52] Liang X., Zhang Z., Yan L., Ding B., Bai J., Yang J., Liu H. (2025). Discrimination and Characterization of Volatile Compounds Variations during Ghee Fermentation Using GC—MS and GC—IMS. Food Biosci..

[53] Cantele C., Potenziani G., Bonciolini A., Bertolino M., Cardenia V. (2024). Effect of Alkylresorcinols Isolated from Wheat Bran on the Oxidative Stability of Minced-Meat Models as Related to Storage. Antioxidants.

[54] Al-Sheikhly M., Poster D.L., An J.-C., Neta P., Silverman J., Huie R.E. (2006). Ionizing Radiation-Induced Destruction of Benzene and Dienes in Aqueous Media. Environ. Sci. Technol..

[55] Guo H., Feng T., Qi W., Kong Q., Yue L., Wang H. (2021). Effects of Electron-beam Irradiation on Volatile Flavor Compounds of Salmon Fillets by the Molecular Sensory Science Technique. J. Food Sci..

[56] Martin D., Joly C., Dupas-Farrugia C., Adt I., Oulahal N., Degraeve P. (2023). Volatilome Analysis and Evolution in the Headspace of Packed Refrigerated Fish. Foods.

[57] Bliznyuk U., Avdyukhina V., Borshchegovskaya P., Bolotnik T., Ipatova V., Nikitina Z., Nikitchenko A., Rodin I., Studenikin F., Chernyaev A. (2022). Effect of Electron and X-Ray Irradiation on Microbiological and Chemical Parameters of Chilled Turkey. Sci. Rep..

[58] Shin W.-G., Ramos-Mendez J., Tran N.H., Okada S., Perrot Y., Villagrasa C., Incerti S. (2021). Geant4-DNA Simulation of the Pre-Chemical Stage of Water Radiolysis and Its Impact on Initial Radiochemical Yields. Phys. Medica.

[59] Sunnerberg J.P., Zhang R., Gladstone D.J., Swartz H.M., Gui J., Pogue B.W. (2023). Mean Dose Rate in Ultra-High Dose Rate Electron Irradiation Is a Significant Predictor for O_2_ Consumption and H_2_O_2_ Yield. Phys. Med. Biol..

[60] Kusumoto T., Danvin A., Mamiya T., Arnone A., Chefson S., Galindo C., Peaupardin P., Raffy Q., Kamiguchi N., Amano D. (2024). Dose Rate Effects on Hydrated Electrons, Hydrogen Peroxide, and a OH Radical Molecular Probe Under Clinical Energy Protons. Radiat. Res..

[61] Mel’nikov M.Y. (2009). Experimental Methods of High-Energy Chemistry.

[62] Krot V.I., Golubeva E.N., Muzyka T.V., Stepanova O.Y. (2011). Chemical Methods of Dosimetry. Ferrosulfate Dosimetry Method (Fricke Dosimeter): Method. Manual for the Workshop.

[63] Chen C., He Y., Wu J. (1998). Selecting Ethanol as a Model Organic Solvent in Radiation Chemistry—3. Radiolysis of Glycyrrhetinic Acid (GL)-Ethanol System and Structure Modification of GL by γ Radiation Method. Radiat. Phys. Chem..

[64] Jin H., Wu J., Pan X., Zhang X. (1996). Selecting Ethanol as a Model Organic Solvent in Radiation Chemistry—I. Radiolysis of Acetone-Ethanol System. Radiat. Phys. Chem..

[65] Jin H., Wu J., Fang X., Zhang X. (1996). Selecting Ethanol as a Model Organic Solvent in Radiation Chemistry—II. γ and Pulse Radiolysis of the Acetophenone-Ethanol System. Radiat. Phys. Chem..

[66] Bliznyuk U., Borshchegovskaya P., Bolotnik T., Chernyaev A., Ipatova V., Nikitchenko A., Shinkarev O., Yurov D., Khmelevskiy O., Rodin I. (2022). Research into Gas Chromatography–Mass Spectrometry (GC-MS) for Ensuring the Effect of 1 MeV-Accelerated Electrons on Volatile Organic Compounds in Turkey Meat. Separations.

[67] Sazonov A.B., Marchenko N.V., Nikitin A.V. (2015). Gamma-Radiolysis of the Ethanol-Water Binary System in the Presence of Oxygen. High Energy Chem..

[68] Chatgilialoglu C., Barata-Vallejo S., Gimisis T. (2024). Radical Reactions in Organic Synthesis: Exploring in-, on-, and with-Water Methods. Molecules.

[69] Ayala A., Muñoz M.F., Argüelles S. (2014). Lipid Peroxidation: Production, Metabolism, and Signaling Mechanisms of Malondialdehyde and 4-Hydroxy-2-Nonenal. Oxid. Med. Cell Longev..

[70] Zhao M., Liu Z., Zhang W., Xia G., Li C., Rakariyatham K., Zhou D. (2025). Advance in Aldehydes Derived from Lipid Oxidation: A Review of the Formation Mechanism, Attributable Food Thermal Processing Technology, Analytical Method and Toxicological Effect. Food Res. Int..

[71] Oyama M. (1965). A Free-Radical Reaction of Primary and Secondary Alcohols with Formaldehyde. J. Org. Chem..

[72] Zhao L., Mi D., Hu B., Sun Y. (2015). A generalized target theory and its applications. Sci. Rep..

[73] Kudryashov Y.B. (2004). Radiation Biophysics (Ionizing Radiation).

[74] Zhao L., Chen X., Tian J., Shang Y., Mi D., Sun Y. (2020). Generalized Multi-Hit Model of Radiation-Induced Cell Survival with a Closed-Form Solution: An Alternative Method for Determining Isoeffect Doses in Practical Radiotherapy. Radiat. Res..

[75] Spinks J.W.T., Woods R.J. (1964). An Introduction to Radiation Chemistry.

[76] Schuler R.H., Allen A.O. (1956). Yield of the Ferrous Sulfate Radiation Dosimeter: An Improved Cathode-Ray Determination. J. Chem. Phys..

[77] O’Leary M., Boscolo D., Breslin N., Brown J.M.C., Dolbnya I.P., Emerson C., Figueira C., Fox O.J.L., Grimes D.R., Ivosev V. (2018). Observation of Dose-Rate Dependence in a Fricke Dosimeter Irradiated at Low Dose Rates with Monoenergetic X-Rays. Sci. Rep..

[78] Spectrophotometer UV-3000. https://istina.msu.ru/equipment/card/615320740/.

[79] Perdew J.P., Burke K., Ernzerhof M. (1996). Generalized Gradient Approximation Made Simple. Phys. Rev. Lett..

[80] Laikov D.N. (2020). Optimization of atomic density-fitting basis functions for molecular two-electron integral approximations. J. Chem. Phys..

[81] Laikov D.N., Ustynyuk Y.A. (2005). PRIRODA-04: A quantum-chemical program suite. New possibilities in the study of molecular systems with the application of parallel computing. Russ. Chem. Bull..

